# Curcumin interacts with sildenafil to kill GI tumor cells via endoplasmic reticulum stress and reactive oxygen/ nitrogen species

**DOI:** 10.18632/oncotarget.19807

**Published:** 2017-08-02

**Authors:** Jane L. Roberts, Andrew Poklepovic, Laurence Booth

**Affiliations:** ^1^ Departments of Biochemistry and Molecular Biology, Virginia Commonwealth University, Richmond, VA 23298-0035, USA; ^2^ Departments of Biochemistry and Medicine, Virginia Commonwealth University, Richmond, VA 23298-0035, USA

**Keywords:** sildenafil, autophagy, chaperone, death receptor, ER stress

## Abstract

The present studies focused on the ability of the phosphodiesterase 5 (PDE5) inhibitor sildenafil to enhance the anti-cancer properties of clinically relevant concentrations of the dietary diarylheptanoid curcumin. In gastrointestinal tumor cells, sildenafil and curcumin interacted in a greater than additive fashion to kill. Inhibition of the extrinsic apoptotic pathway suppressed killing by ∼50%, as did blockade of the intrinsic apoptotic pathway. Sildenafil and curcumin reduced mTORC1 and mTORC2 activity and increased Beclin1 levels and the numbers of autophagosomes and autolysosomes in cells in a PERK-eIF2α-dependent fashion. Knock down of Beclin1 or ATG5 partially suppressed killing. In contrast, stable knock out of ATG16-L1 unexpectedly enhanced killing, an effect not altered by Beclin1/ATG5 knock down. Curcumin and sildenafil exposure reduced the expression of MCL-1, BCL-XL, thioredoxin and superoxide dismutase 2 (SOD2) in an eIF2α-dependent fashion. Curcumin and sildenafil interacted in a greater than additive fashion to increase the levels of reactive oxygen species; knock down of thioredoxin or SOD2 enhanced killing and over-expression of thioredoxin or SOD2 suppressed killing. *In vivo*, curcumin and sildenafil interacted to suppress the growth of colon cancer tumors. Multiplex analyses of plasma taken after drug exposure at animal nadir indicated that the levels of M-CSF, CXCL-9, PDGF and G-CSF were significantly increased by [curcumin + sildenafil] and that expression of CXCL1 and CCL5 were significantly reduced. Cells isolated from *in vivo* treated [curcumin + sildenafil] tumors were resistant to *in vitro* [curcumin + sildenafil] exposure, a phenotype that was blocked by the colon cancer therapeutic regorafenib.

## INTRODUCTION

Prior studies have demonstrated that phosphodiesterase 5 (PDE5) inhibitors such as sildenafil enhance the anti-cancer properties of the NSAID celecoxib [1]. PDE5 inhibitors also enhance killing by a derivative of celecoxib which does not inhibit COX2, OSU-03012 [2, 3]. Subsequently, ourselves and others have shown that PDE5 inhibitors have anti-cancer properties in combination with multi-kinase inhibitors and can enhance the lethality of standard of care chemotherapeutic agents such as doxorubicin or cisplatin; two clinical trials using sildenafil in combination with other drugs are presently open at VCU Massey Cancer Center (NCT02466802; NCT01817751) [4-8].

PDE5 inhibitors were initially developed to treat high blood pressure but subsequently have been used as an erectile dysfunction therapeutic [9]. Recent studies have shown PDE5 to be over-expressed in hepatoma, breast and NSCLC when compared to matched normal tissues [10]. PDE5 catalyzes the degradation of cyclic GMP (cGMP); cGMP acts to cause activation of protein kinase G (PKG). PKG signaling in a cell-type dependent fashion, via activation of the ERK1/2, p38 MAPK, JNK1/2 and NFκB pathways, enhances inducible nitric oxide synthase (iNOS) expression [11-13]. Elevated iNOS expression increases the levels of nitric oxide (NO) which can in turn feedback onto a heme group in soluble guanylyl cyclase (GC) [14]. GC activation increases cGMP levels which causes further PKG activation. Tumor cells, compared to non-transformed cells, generate high levels of reactive oxygen species, e.g. the superoxide anion (O_2_^-^), likely due to the uncoupling of mitochondrial oxidative phosphorylation [15, 16]. NO reacts with O_2_^-^ and forms the toxic oxidant peroxy-nitrite (ONOO^-^) which chemically inactivates DNA, proteins and lipids [17-21].

Curcumin (diferuloylmethane) is a compound most commonly purified from the roots of the plant *Curcuma longa*. The compound has a number of uses including as a food coloring (E100), an ingredient in cooking and as a herbal-Ayurvedic medicine supplement [22]. The compound can be safely dosed in healthy volunteers at 12 grams QD for up to three months [23]. However, curcumin is poorly soluble in water and undergoes rapid first pass metabolism by the liver resulting in low unsustainable levels in the plasma/tissues outside of GI and enterohepatic system. The majority of pre-clinical studies have used curcumin *in vitro* in the non-physiological range of 10 - 50 μM, which is in contrast to the transient increase in peripheral blood plasma concentration which is ∼0.8 μM, in healthy volunteers ingesting 12 g of the compound [24-31]. The use of non-physiological *in vitro* concentrations of 10 μM or greater, may have resulted in the key targets of the chemical as an anti-cancer agent currently being poorly understood/misinterpreted. For example, curcumin concentrations in the 10-20 μM range alone can generate toxic levels of reactive oxygen and nitrogen species in tumor cells. Furthermore, curcumin has been suggested to act as an HDAC inhibitor and to suppress NFκB and AP-1 signaling; HDAC inhibitors are known to elevate ROS levels [32-34].

The present studies were designed to determine whether curcumin and sildenafil interacted to kill GI tumor cells (colon; liver; stomach), at or close to physiological concentrations of the agent as found in the peripheral vasculature and if so, the mechanisms involved. Previous work has shown that curcumin interacted with the NSAID celecoxib to enhance cell killing of colorectal cancer cells [35]. Thus, we also investigated whether celecoxib could further enhance the cell killing potential of the curcumin and sildenafil combination. The tumor types were selected as those most likely to be amenable in a patient for use of oral curcumin (E100) as a therapeutic.

## RESULTS

**Figure 1:**
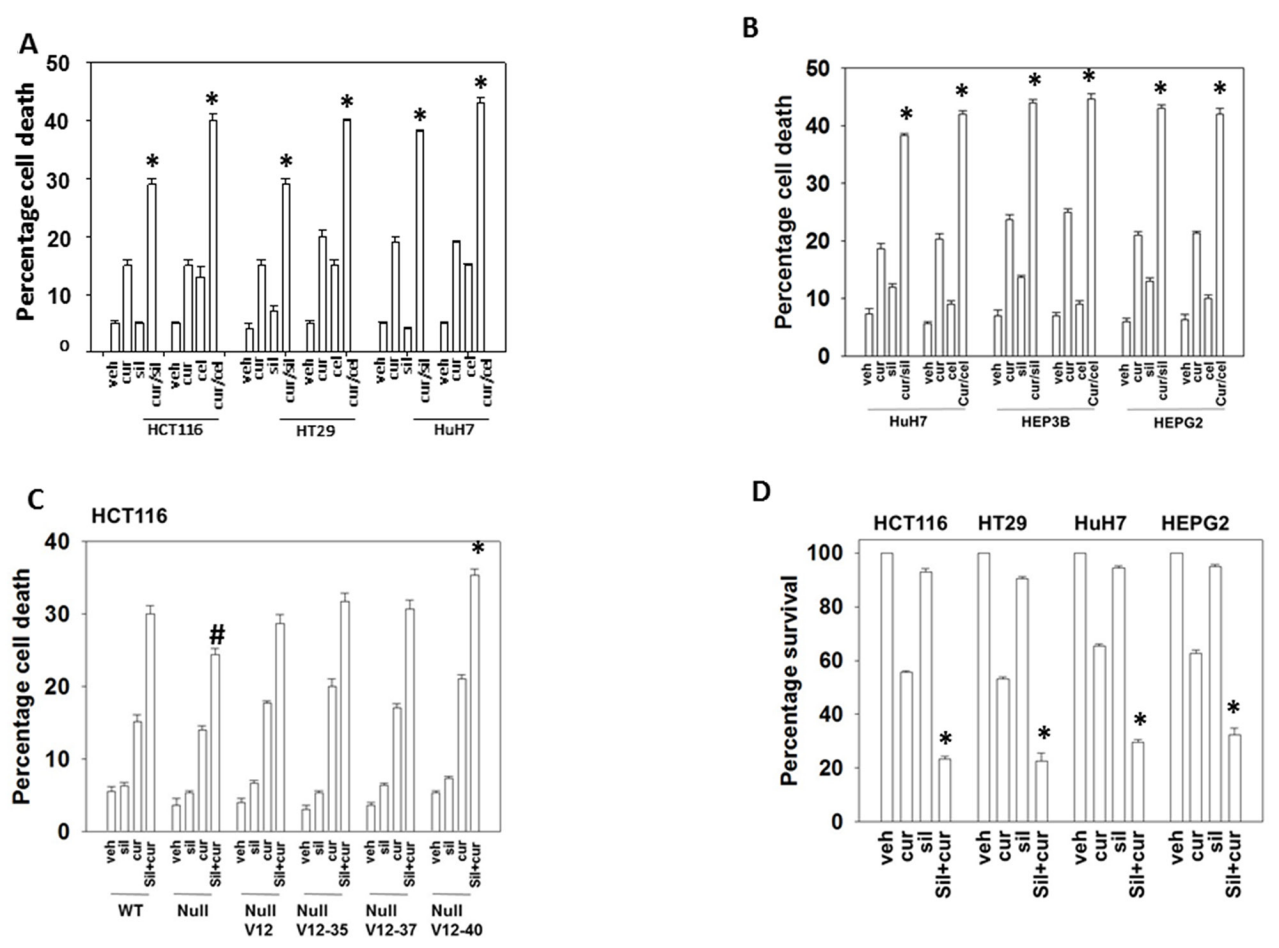
Curcumin interacts with sildenafil and with celecoxib to kill GI tumor cells **(A)** Colon cancer cells and **(B)** liver cancer cells were treated with vehicle control, curcumin (2.0 μM), sildenafil (2.0 μM), celecoxib (2.0 μM) or the drugs in the indicated combinations for 24h. Cell death was measured by trypan blue exclusion (n = 3 +/-SEM) * p < 0.05 greater than individual drug treatments. **(C)** HCT116 cells (parental wild type; K-RAS D13 deleted, C2; C2 cells transfected to express H-RAS V12, C10; C2 cells transfected to express H-RAS V12 C10-35 that activates the ERK1/2 pathway; C2 cells transfected to express H-RAS V12 C10-37 that activates RAL GDS; C2 cells transfected to express H-RAS V12 C10-40 that activates the PI3K pathway) were treated for 12h with vehicle control or with sildenafil (2.0 μM) and/or curcumin (2.0 μM), alone or in combination as indicated. Cell death was measured by trypan blue exclusion (n = 3 +/-SEM) * p < 0.05 greater killing than corresponding value in wild type; ^#^p < 0.05 less killing than corresponding value in wild type. **(D)** Tumor cells as single cells were plated (250-1,000 cells / 60 mm dish). Cells were permitted to attach for 24h after which cells were treated for 24h with vehicle, curcumin (2.0 μM), sildenafil (2.0 μM) or the drugs in combination. After 24h the cells were washed in drug free media and cultured for ∼10 days in drug free media. Colony formation (> 50 cells / colony) was determine for each condition and the plating efficiency determined and plotted (n = 6 +/- SEM). * p < 0.05 less than curcumin single agent.

Curcumin interacted with the PDE5 inhibitor sildenafil or with the NSAID celecoxib to kill multiple GI tumor cell lines within 24h (Figures 1A-1B and Supplementary Figure 1). In HCT116 colon cancer cells that had been genetically manipulated to delete their single allele of K-RAS D13 or in deleted cells engineered to express various forms of H-RAS V12 we found that transformed but non-tumorigenic K-RAS D13 deleted cells were *less sensitive* to the drug combination whereas H-RAS V12 transfected cells which have hyper-activated both the PI3K and ERK1/2 pathways were *more sensitive* to the drugs (Figure 1C). Mutant K-RAS deleted HCT116 cells that expressed H-RAS V12 C40, the H-RAS mutant which specifically activates the PI3K pathway, were *additionally sensitive* to the drug combination comparing to isogenic cells expressing H-RAS V12. Thus, high activity in the ERK1/2 pathway, but especially the PI3K pathway, predicts for a stronger anti-tumor effect following [curcumin + sildenafil] exposure. In colony formation assays, a 24h exposure to curcumin significantly reduced the clonogenicity of liver and colon cancer cells that was itself significantly enhanced by combined exposure with sildenafil (Figure 1D).

**Figure 2:**
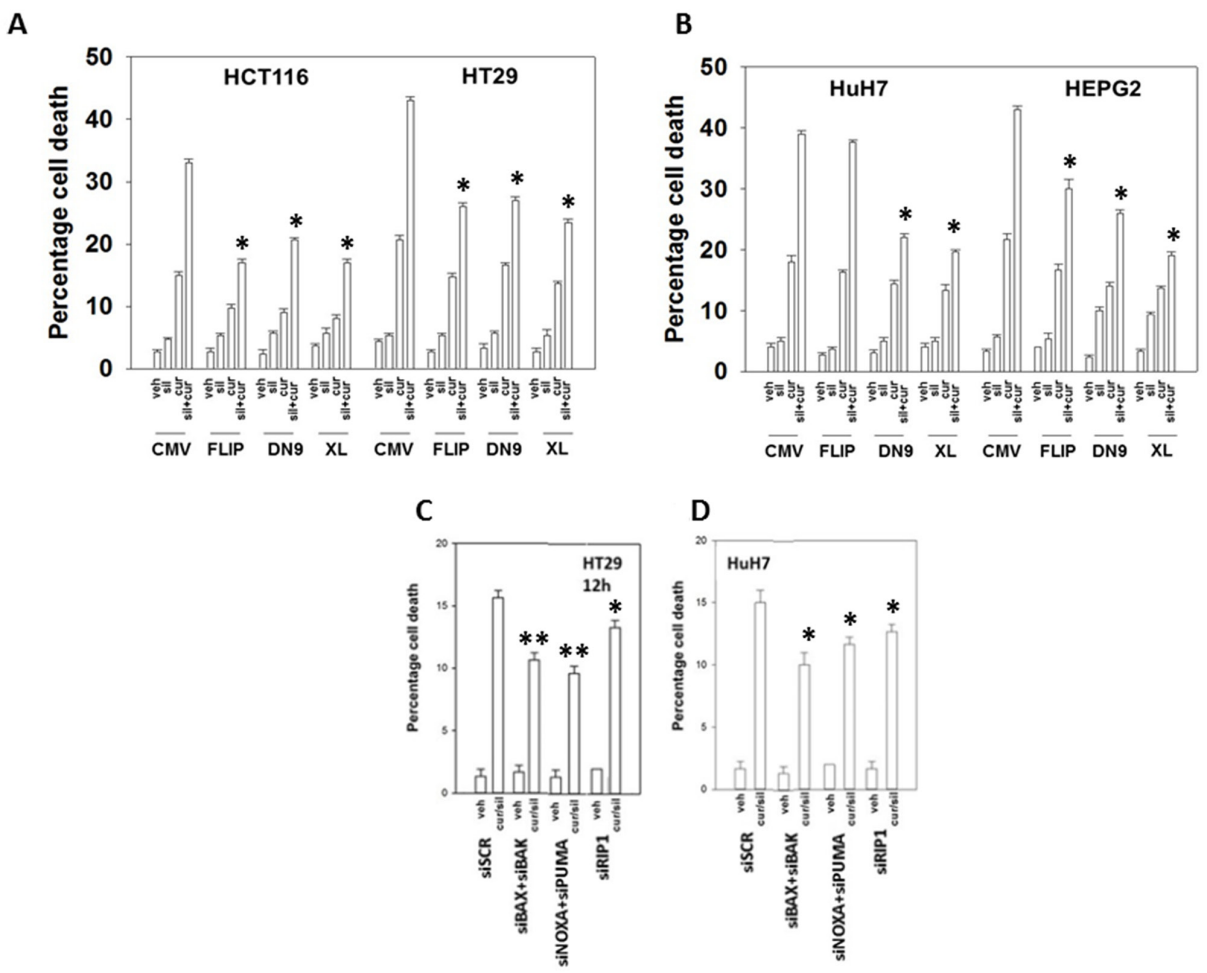
Curcumin and sildenafil kill through activation of the extrinsic and intrinsic pathways Colon cancer cells **(A)** or liver cancer cells **(B)** were transfected with empty vector control CMV plasmid or to express: c-FLIP-s; BCL-XL; or dominant negative caspase 9. Colon cancer cells **(C)** or liver cancer cells **(D)**, were transfected with a scrambled siRNA control (siSCR) or with siRNA molecules to knock down: BAX+BAK; NOXA+PUMA; or RIP1. Twenty-four h after plating cells were treated for 24h with vehicle, curcumin (2.0 μM), sildenafil (2.0 μM) or the drugs in combination. Cell death was measured by trypan blue exclusion (n = 3 +/-SEM) * p < 0.05 less killing than corresponding value in CMV / siSCR transfected; ** p < 0.05 less killing than corresponding value in siRIP-1 cells.

**Figure 3:**
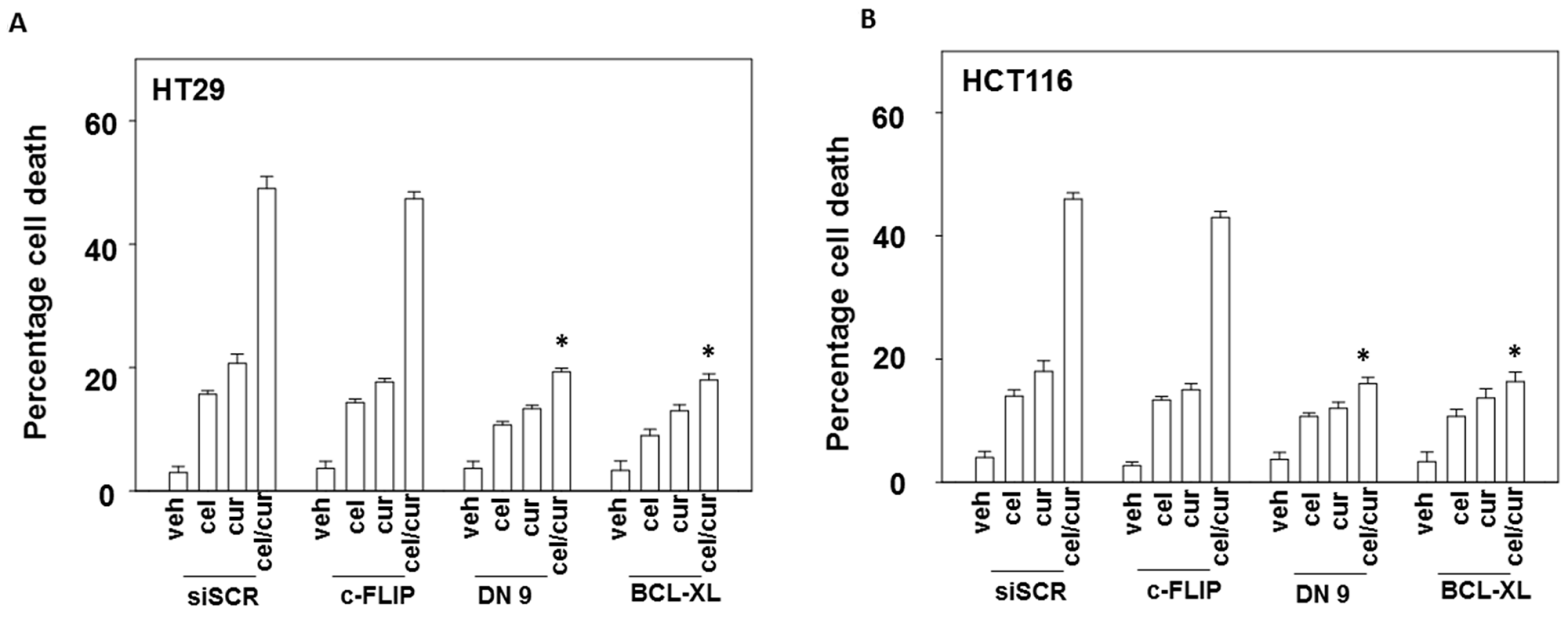
Curcumin and celecoxib interact to kill through the intrinsic pathway **(A-B)** Colon cancer cells were transfected with empty vector control or to express: c-FLIP-s; BCL-XL; or dominant negative caspase 9. Twenty-four h later cells were treated for 24h with vehicle, curcumin (2.0 μM), celecoxib (2.0 μM) or the drugs in combination. Cell death was measured by trypan blue exclusion (n = 3 +/-SEM) * p < 0.05 less killing than corresponding value in CMV transfected.

In HEPG2, HCT116 and HT29 cells that express the death receptor CD95, inhibition of caspase 8 by over-expression of c-FLIP-s, a protein which negatively regulates the signaling complex downstream of death receptors, significantly reduced the lethality of [curcumin + sildenafil] (Figures 2A-2B and Supplementary Figure 2A). Treatment with [curcumin + sildenafil] also increased expression of the death receptors DR4 and DR5, which induce cell death via caspase dependent apoptosis. Expression of DR4 and DR5 was blocked by knock down of eIF2α-CHOP stress signaling (Supplementary Figure 3). HuH7 cells were effectively killed by the drug combination even though they lack expression of the CD95 death receptor and were not protected by the negative regulation of the signaling complex downstream of death receptors (c-FLIPs over expression), (Figure 2B). Knock down of the pro-apoptotic proteins [BAX + BAK] or [NOXA + PUMA] significantly reduced the lethality of [curcumin + sildenafil] treatment (p < 0.01), as did knocking down RIP-1, a protein important in necroptotic cell death [35]. However, knockdown of RIP1 was significantly less protective (p < 0.05) (Figures 2C, 2D and Supplementary Figure 2B). In contrast, for cells treated with the combination of curcumin and celecoxib, death receptor signaling appeared not to be a component of the mechanism (Figure 3A and 3B) [36].

**Figure 4:**
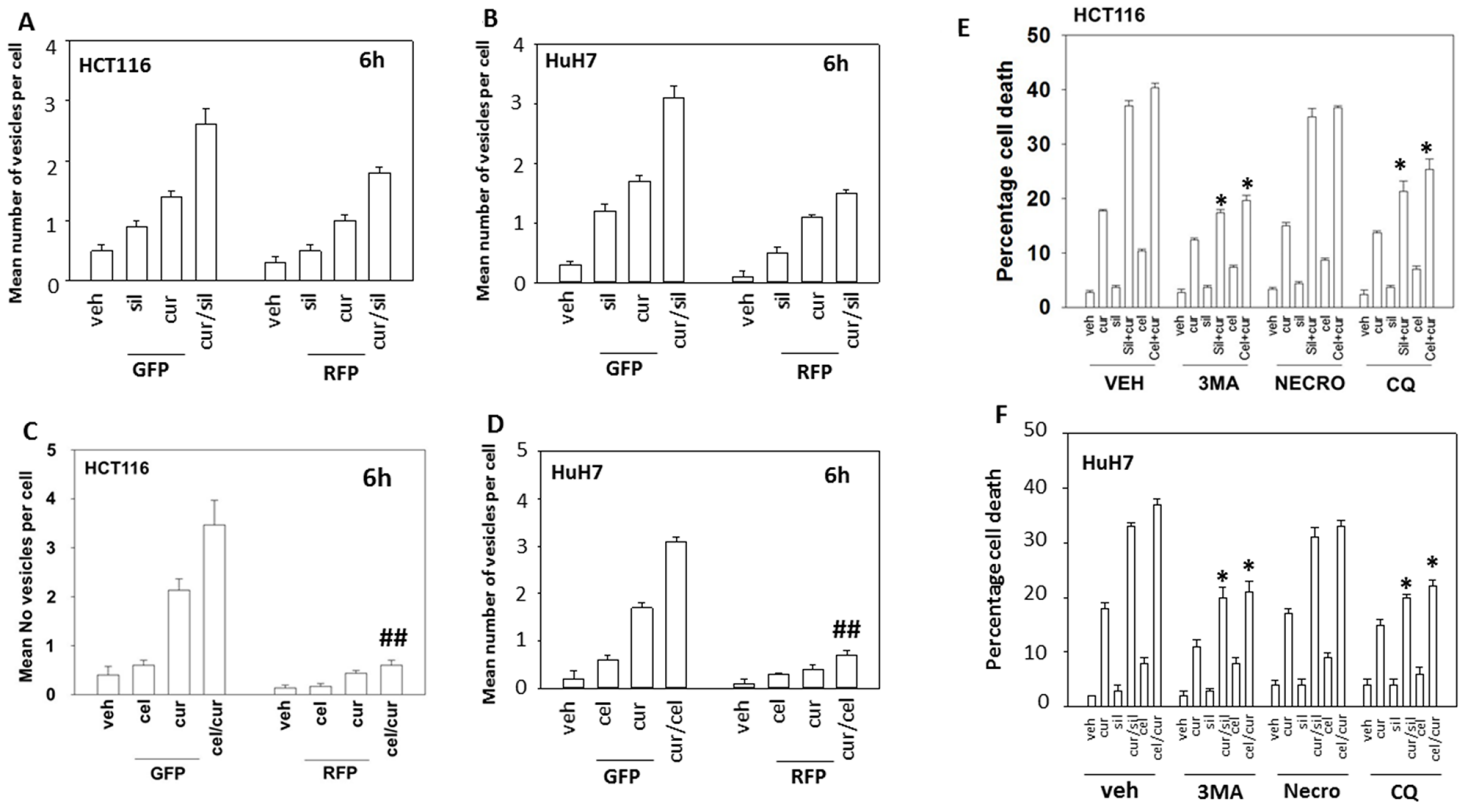
Curcumin and sildenafil increase autophagosome and autolysosome levels that is causal in tumor cell killing **(A)** HCT116 cells and **(B)**, HuH7 cells, were transfected with a plasmid to express LC3-GFP-RFP. Twenty-four h later cells were treated for 6h with vehicle, curcumin (2.0 μM), sildenafil (2.0 μM) or the drugs in combination. At each time point cells were microscopically examined at 40X for the presence of punctate green and red vesicles in at least 40 cells. The mean number of vesicles per cell is plotted (n = 3 +/-SEM). **(C)** HCT116cells and **(D)** HuH7cells were transfected with a plasmid to express LC3-GFP-RFP. Twenty-four h later cells were treated for 6h with vehicle, curcumin (2.0 μM), celecoxib (2.0 μM) or the drugs in combination. At each time point cells were microscopically examined at 40X for the presence of punctate green and red vesicles in at least 40 cells. The mean number of vesicles per cell is plotted (n = 3 +/-SEM). ^##^ p < 0.05 less than corresponding value in cur/sil treated cells in Panels (A) and (B). **(E)** HCT116 cells and **(F)**. HuH7 cells, were treated for 24h with vehicle, curcumin (2.0 μM), sildenafil (2.0 μM), celecoxib (2.0 μM) or the drugs in combination. Where indicated the groups of cells were treated with vehicle control, 3-methyl adenine (10 μM); necrostatin (1 μM) or chloroquine (1 μM). Cell death was measured by trypan blue exclusion (n = 3 +/-SEM) ^*^ p < 0.05 less killing than corresponding value in vehicle.

Treatment of cells with [curcumin + sildenafil] increased the numbers of autophagosomes and subsequently autolysosomes in cells (Figure 4A and 4B). In contrast, treatment of cells with [curcumin + celecoxib] increased the levels of autophagosomes but without strongly increasing autolysosome levels (Figure 4C and 4D). Two inhibitors of autophagy, chloroquine and 3-methyl adenine were both shown to suppress killing by [curcumin + sildenafil] and by [curcumin + celecoxib] (Figure 4E and 4F).

**Figure 5:**
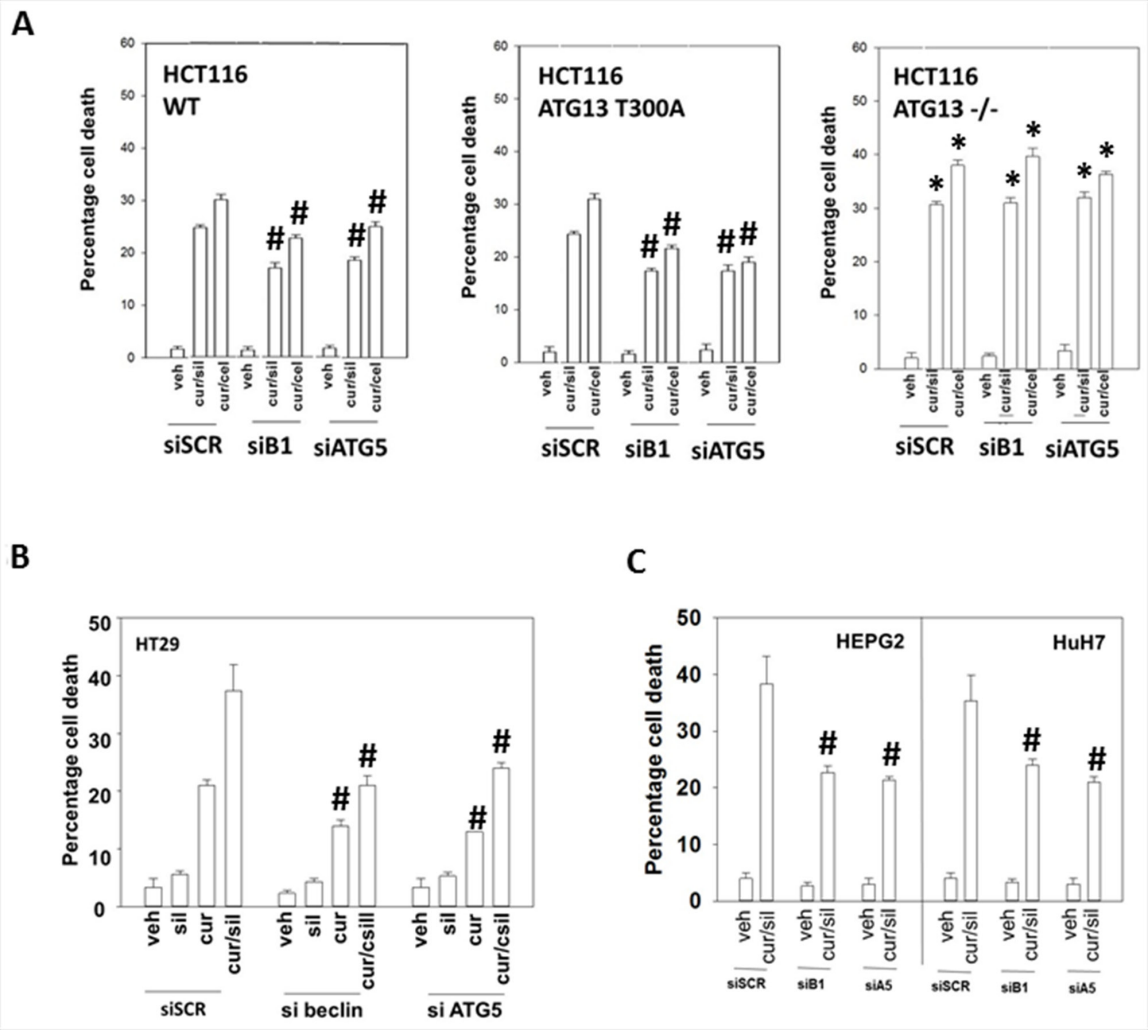
Modulation of autophagy can suppress or enhance [curcumin + sildenafil] lethality based on whether the inhibition of autophagy is transient or stable **(A)** HCT116 cells (wild type; ATG16L1 null -/-; ATG16L1 null cells stably transfected to express ATG16L1 T300A) were treated with vehicle control; with [curcumin (2.0 μM) + sildenafil (2.0 μM)]; or with [curcumin (2.0 μM) + celecoxib (2.0 μM)] for 24h. Cell death was measured by trypan blue exclusion (n = 3 +/-SEM) * p < 0.05 greater killing than corresponding value in HCT116 wild type cells; ^#^ p < 0.05 less killing than in siSCR cells. **(B-C)** GI tumor cells were treated with vehicle control; with [curcumin (2.0 μM) + sildenafil (2.0 μM)] for 24h. Cell death was measured by trypan blue exclusion (n = 3 +/-SEM). ^#^ p < 0.05 less killing than in siSCR cells.

We then made use of HCT116 cells that had been genetically modified with respect to the obligate required autophagy gene ATG16L1 [37]. A threonine 300 to alanine 300 variation in ATG16L1, which has a ∼50% penetrance in European populations but is much less frequently found in African-American populations (∼5%), has been implicated in the development of Crohn’s Disease and an inability of bacteria to be phagocytosed by cells and then disposed of by digestion in autophagic vesicles [38-39]. Wild type HCT116 cells express ATG16L1 T300 T300, which is the isoform of the gene most commonly found in African-Americans. HCT116 ATG16L1 null cells were generated and into those cells was stably transfected a plasmid to code for ATG16L1 T300A, the isoform most commonly found in Caucasians. Both drug combinations caused near-identical levels of cell killing in wild type and T300A HCT116 cells (Figure 5A). This is in contrast to our recent data combining pemetrexed and sorafenib where ATG16L1 T300 T300 cells were more effectively killed than ATG16L1 A300 A300 cells [40]. To our surprise, however, ATG16L1 -/- null cells, which cannot form autophagosomes in response to [curcumin + sildenafil] exposure were more sensitive to both drug combinations, an effect that was not modified by knock down of proteins necessary for autophagy, ATG5 or Beclin1. The autophagy-independent actions of ATG16-L1 are unknown. We then examined the impact of transiently knocking down ATG5 or Beclin1 in other GI tumor cell types. In all the GI cell lines tested in this study, knockdown of either ATG5 or Beclin1 significantly reduced the cell death seen in response to [curcumin + sildenafil] (Figures 5B and 5C; Supplementary Figure 4).

**Figure 6:**
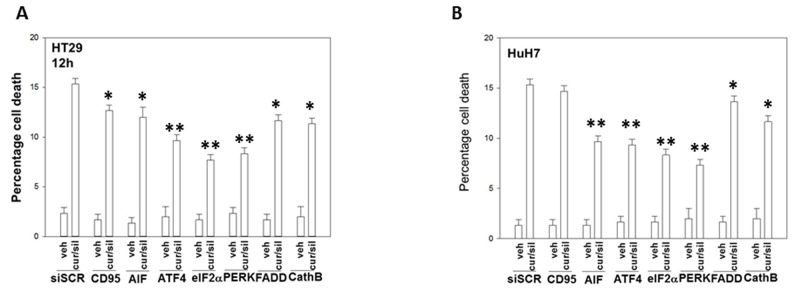
Endoplasmic reticulum stress signaling induced by [curcumin + sildenafil] through PERK-eIF2α causes tumor cell death **(A)** Colon tumor cells and **(B)** liver tumor cells were transfected with a scrambled siRNA control or with siRNA molecules to knock down the expression of: CD95; AIF; ATF4; eIF2α; PERK; FADD; and cathepsin (B). Twenty-four h after transfection cells were treated with vehicle control or with [curcumin (2.0 μM) + sildenafil (2.0 μM)] for 12h. Cell death was measured by trypan blue exclusion (n = 3 +/-SEM). * p < 0.05 less than corresponding value in siSCR cells; ** p < 0.01 less than corresponding value in siSCR cells.

**Figure 7:**
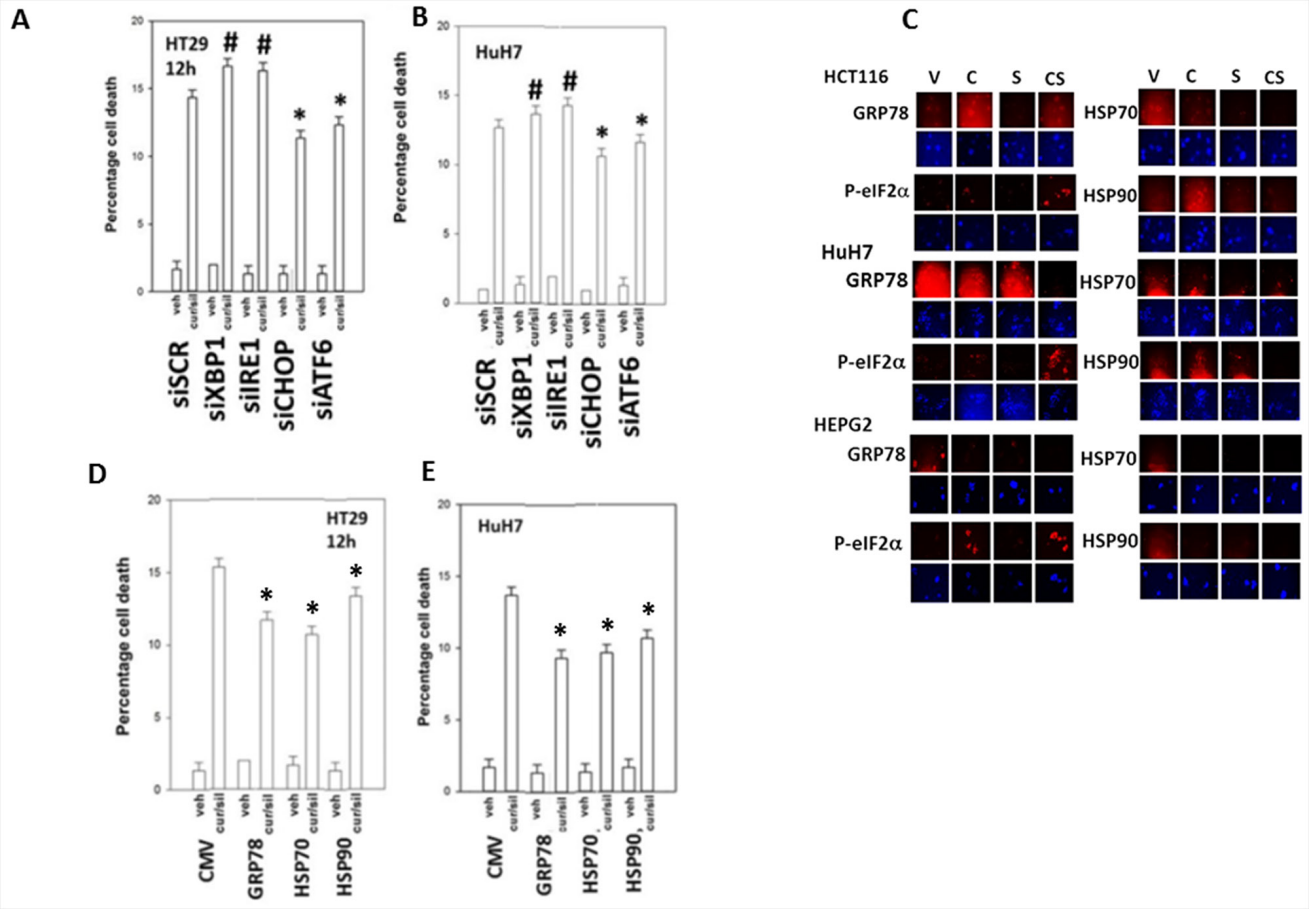
PERK- and ATF6- dependent signaling facilitates [curcumin + sildenafil] toxicity whereas IRE1/XBP1 signaling via chaperone expression is protective **(A-B)** Tumor cells were transfected with a scrambled siRNA control or with siRNA molecules to knock down the expression of: XBP1; IRE1; CHOP; or ATF6. Twenty-four h after transfection cells were treated with vehicle control or with [curcumin (2 μM) + sildenafil (2 μM) for a further 12h. Cell death was measured by trypan blue exclusion (n = 3 +/-SEM). ^#^ p < 0.05 greater than corresponding value in siSCR cells; * p < 0.05 less than corresponding value in siSCR cells. **(C)** Tumor cells were treated with vehicle control; curcumin (2 μM); sildenafil (2 μM) or the drugs in combination for 6h. Cells were fixed in situ and immuno-fluorescence at 10X performed to determine the expression levels of HSP90, HSP70, GRP78 and the serine 51 phosphorylation of eIF2α. **(D-E)** Tumor cells were transfected with an empty vector plasmid (CMV) or plasmids to express: GRP78; HSP90; or HSP70. Twenty-four h after transfection cells were treated with vehicle control or with [curcumin (2 μM) + sildenafil (2 μM) for a further 12h. Cell death was measured by trypan blue exclusion (n = 3 +/-SEM). * p < 0.05 less than corresponding value in siSCR cells.

In prior studies using sildenafil in combination with other agents we have observed the induction of an ER stress response which plays a role in drug combination toxicities. Knock down of one branch of ER stress signaling, PERK-eIF2α-ATF4 signaling, was shown to significantly reduce the lethality of [curcumin + sildenafil] (Figures 6A-6B; Supplementary Figure 5). In congruence with prior data using c-FLIP-s, knock down of CD95/FADD protected the cells as did knock down of the lysosomal protease cathepsin B and apoptosis inducing factor (AIF). In agreement with data in Figure 6, knock down of CHOP, an ER stress induced protein which appears to have a role in the toxicity seen during chronic ER stress [41], significantly suppressed cell killing as did knock down of ATF6, a protein upstream of CHOP, (Figures 7A-7B; Supplementary Figure 6C). In contrast to the PERK / ATF6 branches of ER stress signaling, knock down of IRE1/XBP1 branch of ER stress signaling enhanced drug combination lethality. Treatment of GI tumor cells with [curcumin + sildenafil] reduced the expression of HSP90 and HSP70 in all cells tested and caused an endoplasmic reticulum stress response, as judged by elevated eIF2α phosphorylation (Figure 7C). Of note, in HCT116 cells [curcumin + sildenafil] was shown to enhance GRP78 expression, which would predict for reduced phosphorylation of eIF2α, that eIF2α phosphorylation was increased under these conditions argues that [curcumin + sildenafil] is inactivating protein phosphatase 1 complexed with eIF2α. Over-expression of the chaperone proteins GRP78, HSP90 or HSP70 as individual agents was shown to partially though significantly reduce drug-induced cell killing (Figures 7D-7E; Supplementary Figure 6B).

**Figure 8:**
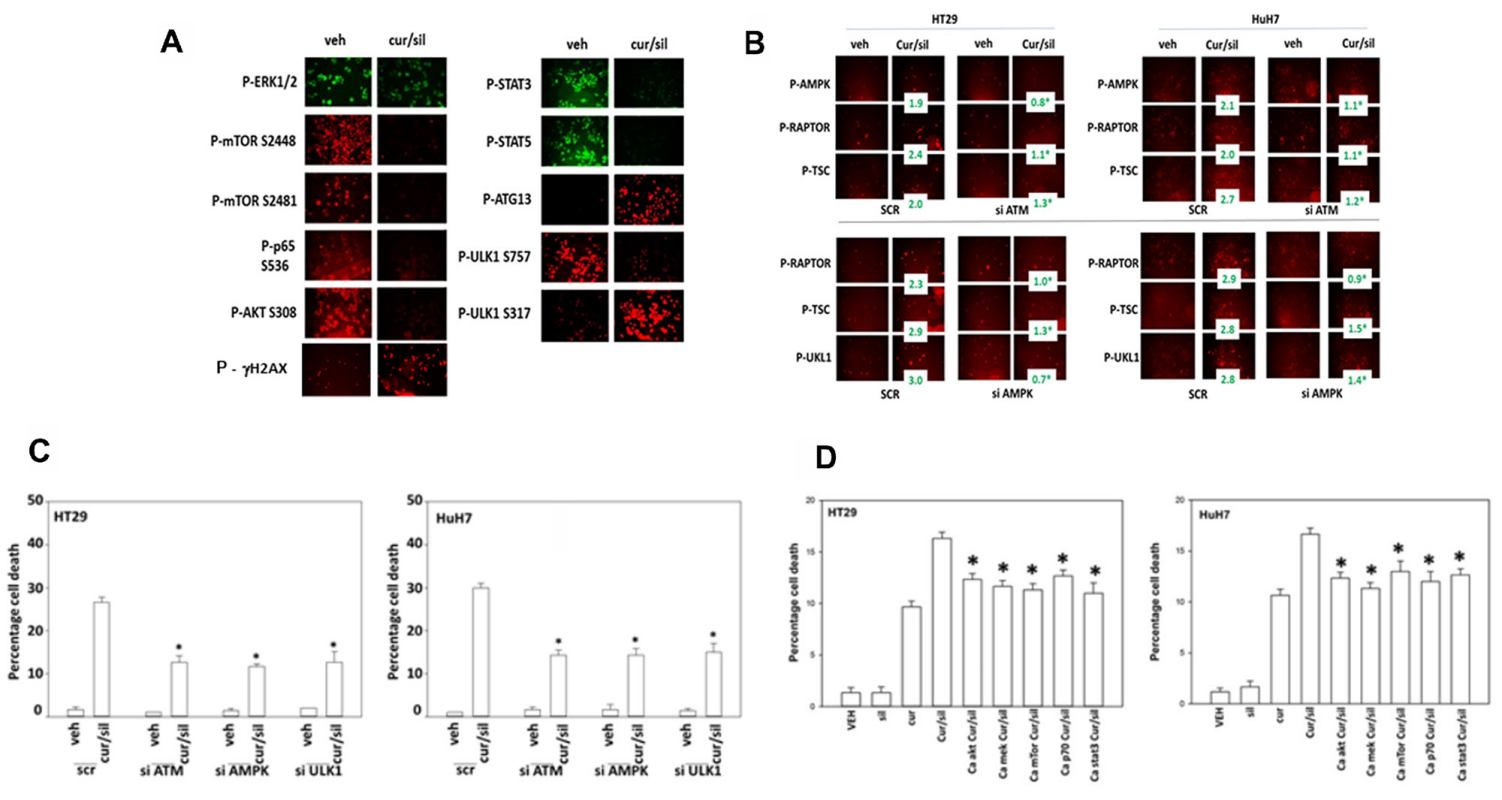
[Curcumin + sildenafil] treatment inactivates multiple cyto-protective signaling pathways **(A)** Tumor cells were treated with vehicle control or with [curcumin (2 μM) + sildenafil (2 μM) for a further 6h. Cells were fixed in place and immuno-fluorescence performed to determine the expression and/or phosphorylation of the indicated proteins. Cells were visualized in a Hermes wide field microscope at 10X magnification. **(B) Upper:** Tumor cells were transfected with a scrambled siRNA (siSCR) or an siRNA to knock down expression of ATM. Twenty-four h after transfection cells were treated with vehicle control or with [curcumin (2 μM) + sildenafil (2 μM) for a further 6h. Cells were fixed in place and immuno-fluorescence performed to determine the expression and/or phosphorylation of the indicated proteins. (n = 3 +/-SEM) * p < 0.05 less than corresponding value in SCR cells. **Lower:** Tumor cells were transfected with a scrambled siRNA (siSCR) or siRNA to knock down expression of AMPKα. Twenty-four h after transfection cells were treated with vehicle control or with [curcumin (2 μM) + sildenafil (2 μM) for a further 6h. Cells were fixed in place and immuno-fluorescence performed to determine the expression and/or phosphorylation of the indicated proteins. (n = 3 +/-SEM) * p < 0.05 less than corresponding value in SCR cells. **(C)** Tumor cells were transfected with a scrambled siRNA (siSCR) or siRNA to knock down expression of AMPKα, ATM or ULK-1. Twenty-four h after transfection cells were treated with vehicle control or with [curcumin (2 μM) + sildenafil (2 μM) for a further 24h. Cell death was measured by trypan blue exclusion (n = 3 +/-SEM) * p < 0.05 less than corresponding value in SCR cells. **(D)** Tumor cells were transfected with an empty vector plasmid (CMV) or plasmids to express: activated AKT; activated MEK1; activated mTOR; activated p70 S6K; and activated STAT3. Twenty-four h after transfection cells were treated with vehicle control or with [curcumin (2 μM) + sildenafil (2 μM) for a further 12h. Cell death was measured by trypan blue exclusion (n = 3 +/-SEM). * p < 0.05 less than corresponding value in CMV cells.

We next examined the changes in survival-regulatory signal transduction pathway activities caused by the [curcumin + sildenafil] combination. Treatment of cells with [curcumin + sildenafil] was shown to rapidly inactivate the activity of AKT, mTORC1, mTORC2, ERK1/2, STAT3, STAT5 (Figure 8A). Expression of activated STAT3 and to a lesser extent activated mTOR prevented the [curcumin + sildenafil]-induced reductions in cell survival protein such as MCL-1, BCL-XL, SOD2, TRX and c-FLIP-s expression (Supplementary Figure 7). Expression of activated STAT3 or activated mTOR also suppressed the drug-induced elevated phosphorylation of the autophagy protein ATG13, the ER stress protein eIF2α, and the elevated expression of CHOP (Supplementary Figure 7). Inhibition of eIF2α–dependent stress signaling was shown to prevent [curcumin + sildenafil]-induced reductions in cell survival protein such as MCL-1, BCL-XL, SOD2, TRX, (S8-S9). Further, Inhibition of eIF2α signaling was shown to abolish the drug-induced expression of the autophagy protein Beclin1 and prevented ATG13 S318 phosphorylation (Supplementary Figure 10). Knock down of eIF2α expression only partially blocked the drug induced inactivation of mTORC1 and mTORC2 (Supplementary Figure 10).

The [curcumin + sildenafil] combination was shown to induce auto-phosphorylation of ataxia-telangiectasia mutated (ATM), an apical activator of the DNA damage response in the face of DNA double-strand breaks, and elevated γ-H2AX phosphorylation, in addition to the activation of the AMP dependent protein kinase (AMPK), as judged by increased threonine 172 phosphorylation of AMPK (Figure 8A and 8B). The drug combination was shown to activate ULK-1, this was indicated by an increased phosphorylation of S318 of ATG13. Further, a decrease in the phosphorylation of ULK-1 at S757 suggested that mTORC1/mTORC2 were both inactivated and the increased phosphorylation of ULK-1 at S317 suggested that AMPK was being activated, which is congruent with our AMPK T172 phosphorylation data.

ATM has been shown to phosphorylate AMPK T172 [23]. In the current study knock down of ATM prevented [curcumin + sildenafil] treatment from altering the phosphorylation of AMPK, RAPTOR and TSC2 [42] (Figure 8B). In addition, knock down of AMPKα prevented [curcumin + sildenafil] treatment from altering the phosphorylation of RAPTOR, TSC2 and ULK-1 S317 (Figure 8B). Knock down of ATM, AMPKα or ULK-1 reduced the ability of [curcumin + sildenafil] to induce autophagosome formation and tumor cell death (Figure 8C; Supplementary Figure 11A). Thus an ATM-AMPK-ULK-1-autophagy regulatory pathway plays a key role in mediating the lethality of [curcumin + sildenafil]. Expression of activated forms AKT, MEK, mTOR, p70 and STAT3 in the GI tumor cells used in this study each showed a significant partial protection of cells from [curcumin + sildenafil] induced cell death, abolishing the cell-death potentiating effect of sildenafil (Figure 8D; Supplementary Figure 11B).

**Figure 9:**
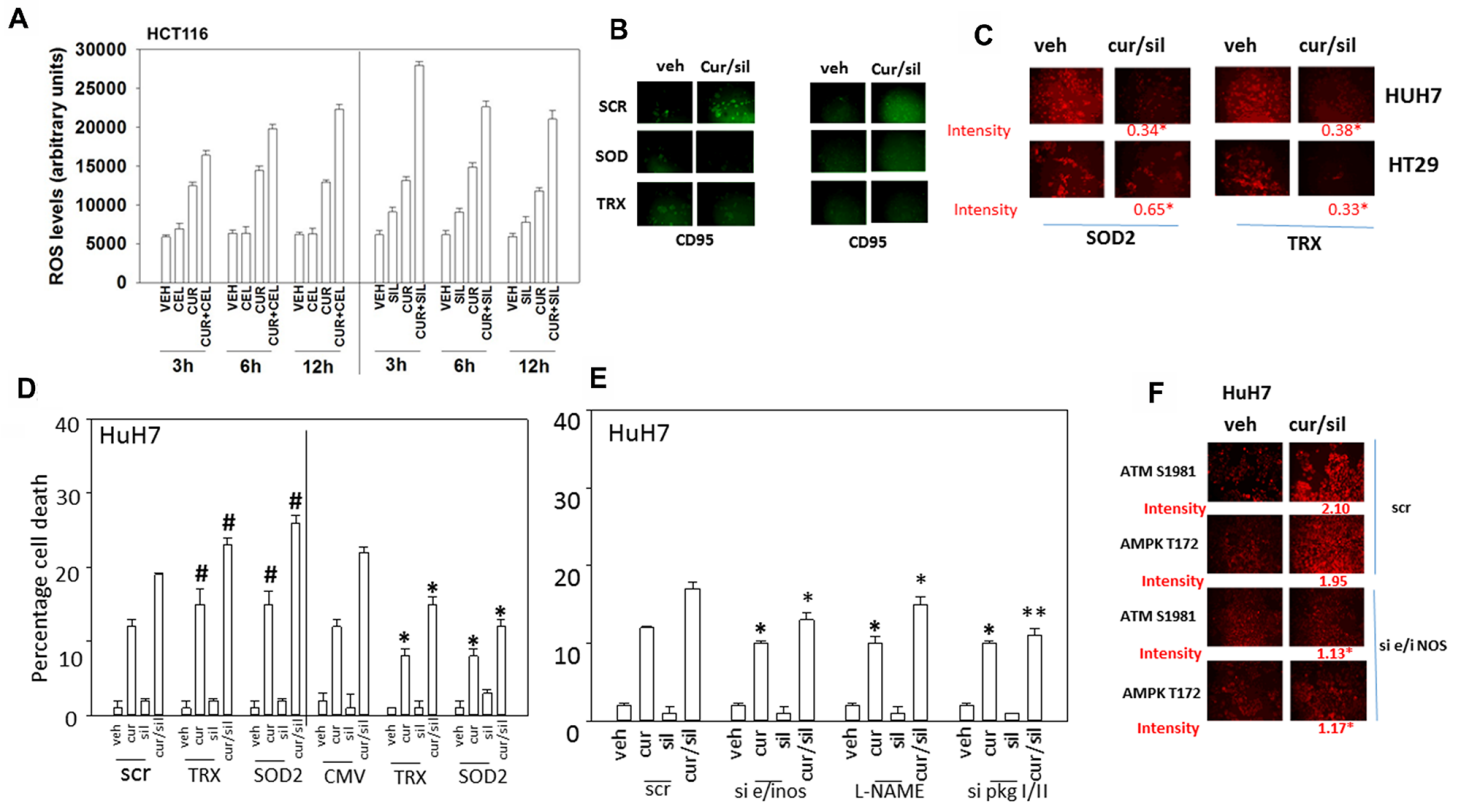
Reactive oxygen / nitrogen species and the suppression of their detoxification by [curcumin + sildenafil] treatment plays a major role in drug combination toxicity **(A)** HCT116 cells were treated with vehicle control, curcumin (2 μM), sildenafil (2 μM) or celecoxib (2 μM) alone or in combination as indicated for the times as shown in the graph (3h, 6h, 12h). Fifteen min prior to assessment of ROS levels, HCT116 cells were pre-incubated with the dye DCFH-DA (5 μM). At each time point cells were placed into a Vector 3 plate reader and the fluorescence of converted DCFH-DA measured (n = 3 +/-SEM). * p < 0.05 greater than corresponding value in cells treated with [curcumin + celecoxib]. **(B)** Tumor cells were transfected with an empty vector plasmid (CMV) or with plasmid to express either TRX or SOD2. Twenty-four h after transfection cells were treated with vehicle control or with [curcumin (2 μM) + sildenafil (2 μM)] in combination for 2h. Cells were fixed in place and not permeabilized. Immuno-staining was performed to determine the total cell surface fluorescence of CD95 staining under each condition (n = 3 +/- SEM). **(C)** Tumor cells were treated with vehicle control, curcumin (2 μM), sildenafil (2 μM) alone or in combination for 6h. Cells were fixed in place and immuno-fluorescence performed to determine the expression of SOD2 and TRX, and the phosphorylation of ATM, AMPK and eIF2α (n = 3 +/-SEM) * p < 0.05 less than value in VEH cells. **(D)** Tumor cells were either transfected with a scrambled siRNA (siSCR) or siRNA molecules to knock down expression of SOD2 or TRX, alternatively other portions of cells were transfected with an empty vector plasmid (CMV) or with plasmids to express SOD2 or TRX. Twenty-four h after transfection cells were treated with vehicle control or [curcumin (2 μM) + sildenafil (2 μM)] in combination for 24h. Cell death was measured by trypan blue exclusion (n = 3 +/-SEM). * p < 0.05 less than corresponding value in CMV cells; ^#^ p < 0.05 greater than corresponding value in siSCR cells. **(E)** Tumor cells were transfected were either transfected with a scrambled siRNA (siSCR) or siRNA molecules to knock down expression of: [iNOS + eNOS] or [PKGI + PKGII]. Twenty-four h after transfection cells were treated with vehicle control or [curcumin (2 μM) + sildenafil (2 μM)] in combination for 24h. Cell death was measured by trypan blue exclusion (n = 3 +/-SEM). * p < 0.05 less than corresponding value in CMV cells; ** p < 0.05 less than corresponding value in L-NAME treated cells. **(F)** Tumor cells were transfected either with a scrambled siRNA (siSCR) or siRNA molecules to knock down expression of [iNOS + eNOS]. Tumor cells were treated with vehicle control or [curcumin (2 μM) + sildenafil (2 μM)] alone or in combination for 6h. Cells were fixed in place and immuno-fluorescence performed to determine the phosphorylation of ATM S1981 and AMPK T172 (n = 3 +/-SEM) * p < 0.05 less than corresponding value in SCR cells.

It has been reported over many years that curcumin can increase the levels of reactive oxygen and reactive nitrogen species in tumor cells [23-24]. Treatment of HCT116 cells with [curcumin + sildenafil] caused a greater than additive increase in the levels of ROS in tumor cells that was of a greater prolonged intensity than cells treated with [curcumin + celecoxib] (Figure 9A). We have previously shown that knock down of CD95 or FADD, or over-expression of c-FLIP-s protected tumor cells from [curcumin + sildenafil] lethality. Treatment with [curcumin + sildenafil] increased cell surface levels of CD95, indicative that CD95 was being activated (Figure 9B). Expression of either SOD2 or TRX was shown to reduce the activation of CD95. Treatment of GI tumor cells with [curcumin + sildenafil] decreased expression of SOD2 and TRX (Figure 9C). Knock down of either TRX or SOD2 expression enhanced [curcumin + sildenafil] lethality whereas over-expression of TRX or SOD2 suppressed drug combination lethality (Figure 9D). Knock down of iNOS and eNOS or treatment of the cells with the pan-NOS inhibitory compound L-NAME suppressed killing by [curcumin + sildenafil] (Figure 9E). In Figure 8A we noted that ULK-1 S317 became phosphorylated after [curcumin + sildenafil] exposure via the AMPK. AMPK T172 phosphorylation and ATM S1981 phosphorylation were increased by [curcumin + sildenafil] in an NOS-dependent fashion (Figure 9F). Prior studies by have shown that nitric oxide, via ATM activation, can increase AMPK T172 phosphorylation [42].

**Figure 10:**
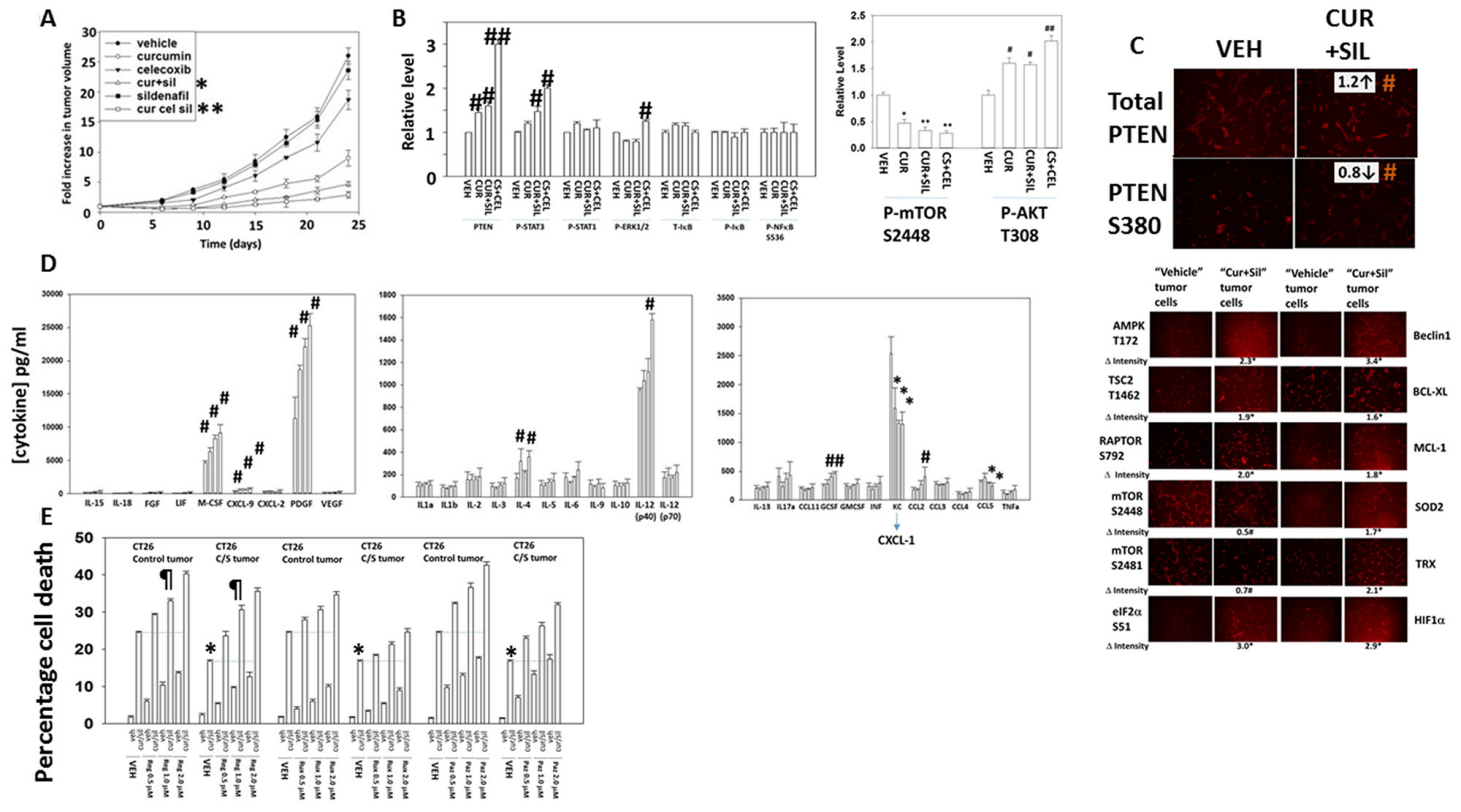
Curcumin combines with sildenafil to reduce tumor growth, an effect that is enhanced by celecoxib **(A)** CT29 cells were implanted into the rear flank of mice and tumors permitted to form (∼45 mm^3^). Animals were grouped with the mean tumor volume of each group being very similar. Animals were then treated for 5 days with curcumin, sildenafil and/or celecoxib as described in the Methods. Tumors were callipered and tumor volumes determined before and following drug exposure (n = 10 +/- SEM) * p < 0.05 less than curcumin alone; ** p < 0.05 less than [curcumin + sildenafil]. **(B)** At the time of animal nadir when tumor volumes were approaching 1,500 mm^3^, tumors were isolated, homogenized and processed according to manufacturer’s instructions to determine the expression and phosphorylation of the indicated proteins. ^#^ p < 0.05 greater than corresponding value in vehicle control cells; ^##^ p < 0.05 greater than value in cur+sil material; * p < 0.05 less than vehicle control value; ** p < 0.05 less than value in curcumin only treated cells. **(C)** CT26 cells isolated from vehicle control treated tumors and [curcumin + sildenafil] treated tumors were plated and 24h later fixed in situ. Immunofluorescence was performed in each isolate to determine the total expression of PTEN and the phosphorylation of PTEN S380. ^#^ p < 0.05 greater mol/mol phosphorylation. **(D)** At the time of animal nadir when tumor volumes were approaching 1,500 mm^3^, mouse plasma was isolated and processed according to manufacturer’s instructions to determine the plasma levels of human cytokines. * p < 0.05 less than corresponding value in vehicle control treated animals; ^#^ p < 0.05 greater than corresponding value in vehicle control cells. **(E)** CT26 cells isolated from vehicle control treated tumors and [curcumin + sildenafil] treated tumors were plated and 24h later were treated with vehicle control, [curcumin (2.0 μM) + sildenafil (2.0 μM)], regorafenib (0-2.0 μM), pazopanib (0-2.0 μM), ruxolitinib (0-2.0 μM) or the drugs in combination as indicated for 12h. Cell death was measured by trypan blue exclusion (n = 3 +/-SEM). * p < 0.05 less than corresponding value in control tumor cells; ¶ p > 0.05 compared to corresponding value in control tumor cells.

Finally, we determined whether the anti-tumor effects of [curcumin + sildenafil] translated into a syngeneic mouse model system. Curcumin and sildenafil interacted to suppress the growth of CT26 mouse colon cancer tumors in their syngeneic immune-competent BALB/c host mouse (Figure 10A). The anti-tumor effect of [curcumin + sildenafil] was magnified by co-exposure of the tumors to the NSAID celecoxib. At the time of mouse nadir, where tumors were > 1,500 mm^3^ in volume, the plasma and tumor from each mouse was isolated and these materials subjected to multiplex antibody array assays in a Bio-Rad MAGPIX system. In tumors previously treated with [curcumin], [curcumin + sildenafil] or [curcumin + sildenafil + celecoxib] the activity of mTOR was reduced and the activity of STAT3 and of AKT was enhanced (Figure 10B). The basal expression level of PTEN was also enhanced and the inhibitory phosphorylation of PTEN at serine 380 was decreased. However, no changes in the basal activities of ERK1/2, NFκB or STAT1 were observed (Figure 10B and 10C). The plasma concentrations of M-CSF, CXCL-9, PDGF and G-CSF were significantly increased and the plasma concentrations of CXCL1 and CCL5 were significantly reduced in animals following either [curcumin] or [curcumin + sildenafil] treatment (Figure 10D). No significant changes in cytokine levels were noted in the plasma from animals treated with [sildenafil] or [celecoxib] (data not shown). In animals, previously exposed to the three drug [curcumin + sildenafil + celecoxib] combination, a similar pattern of changes in cytokine levels was noted when compared to the [curcumin + sildenafil] data, with the exception that IL-12 (p40) plasma levels were profoundly enhanced, as well as a small increase in CCL2 levels.

In Figure 10D the most striking observation was that [curcumin], [curcumin + sildenafil], and [curcumin + sildenafil + celecoxib] all significantly enhanced the plasma levels of PDGF in animals previously treated with the drugs. It would be predicted in a colon cancer patient that elevated PDGF signaling in the long-term would counteract any anti-tumor effects of the drug combinations. The drug regorafenib is approved for late stage colorectal cancer patients; regorafenib inhibits RAF-1, B-RAF as well as PDGFRα/β [42, 43]. In CT26 cells isolated from tumors previously exposed to vehicle control, regorafenib enhanced the lethality of [curcumin + sildenafil] (Figure 10E). In CT26 cells isolated from tumors previously exposed to [curcumin + sildenafil], the killing power of [curcumin + sildenafil] was reduced compared to cells from vehicle control treated tumors. However, the use of regorafenib in combination with [curcumin + sildenafil] largely abolished this reduction in cell killing with no statistically significant difference between the groups using 1.0 μM regorafenib. The drug pazopanib, in contrast to regorafenib, also inhibits PDGFRα/β but does not inhibit RAF-1 or B-RAF. Pazopanib also enhanced [curcumin + sildenafil] lethality, though was less effective than regorafenib suggesting the RAF-1/B-RAF inhibitory properties of regorafenib may also play a role in enhancing killing.

**Figure 11:**
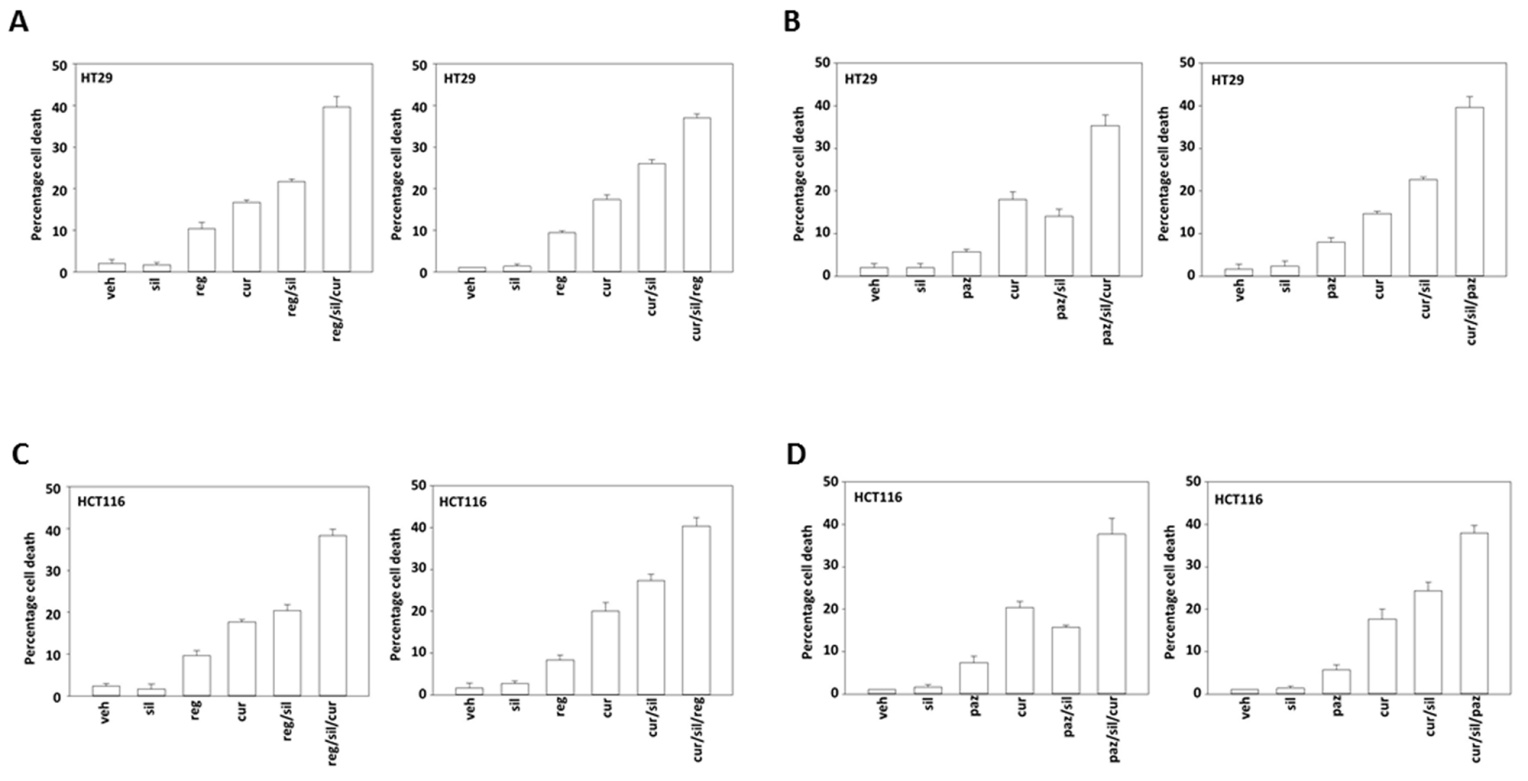
Regorafenib and pazopanib both enhance [curcumin + sildenafil] lethality in colon cancer cells **(A-D)** Colon cancer cell lines were treated with vehicle control, [curcumin (2.0 μM) + sildenafil (2.0 μM)], regorafenib (1.0 μM), pazopanib (1.0 μM) or the drugs in combination as indicated in each graph for 12h. Cell death was measured by trypan blue exclusion (n = 3 +/-SEM).

In adapted / resistant CT26 cells STAT3, but not STAT1 or SRC, was activated. Treatment of adapted CT26 cells with the JAK1/2 inhibitor ruxolitinib modestly enhanced [curcumin + sildenafil] lethality (Figure 10E). This suggests that JAK1/2 were not responsible for STAT3 activation in the resistant cells. Thus, multiplex analyses of plasma and tumor material following [curcumin + sildenafil] exposure can identify the cytoprotective evolutionary pathways which have been selected by drug exposure to maintain tumor cell survival. Similar cell killing data to that in Figure 10E were obtained in human tumor cells when cells were treated with [curcumin + sildenafil] in combination with either pazopanib or regorafenib (Figures 11A-11D; Supplementary Figure 12).

## DISCUSSION

The present studies were designed to determine whether a very low clinically achievable concentration of curcumin could have its anti-cancer properties enhanced by the PDE5 inhibitor sildenafil or by the NSAID celecoxib. Our initial hypotheses were proven correct with all GI tumor cell lines tested in this study responding to the drug combination. Curcumin, derived from the spice turmeric, is an approved food additive (E100) which thus could be used in any future clinical translational studies. There are other clinically tested more soluble and efficacious formulations of curcumin that are commercially available, e.g. Meriva™, CuraPro™, although these formulations are not prepared under GMP conditions which likely prevents their use in clinical trials [43, 44]. For example, whilst a 12 g ingestion of pure curcumin only resulted in a transient peripheral plasma curcumin C max of ∼0.8 μM, a 2-gram ingestion of CuraPro / BCM-95 in healthy human volunteers produced a C max of 1.2 μM after ∼4h and an AUC of 3201 ng/ml/h [43]. Our studies have specifically focused on GI tumors, and within the enterohepatic system and liver the steady state concentration of curcumin, using pure curcumin, is approximately 3 μM. A 400 mg ingestion of celecoxib and a 100 mg ingestion of sildenafil both result in drug C max values of approximately 2 μM. Collectively, our data are supportive of clinical translation of the combination of curcumin, sildenafil and celecoxib using clinically achievable and safe doses of the three agents.

**Figure 12:**
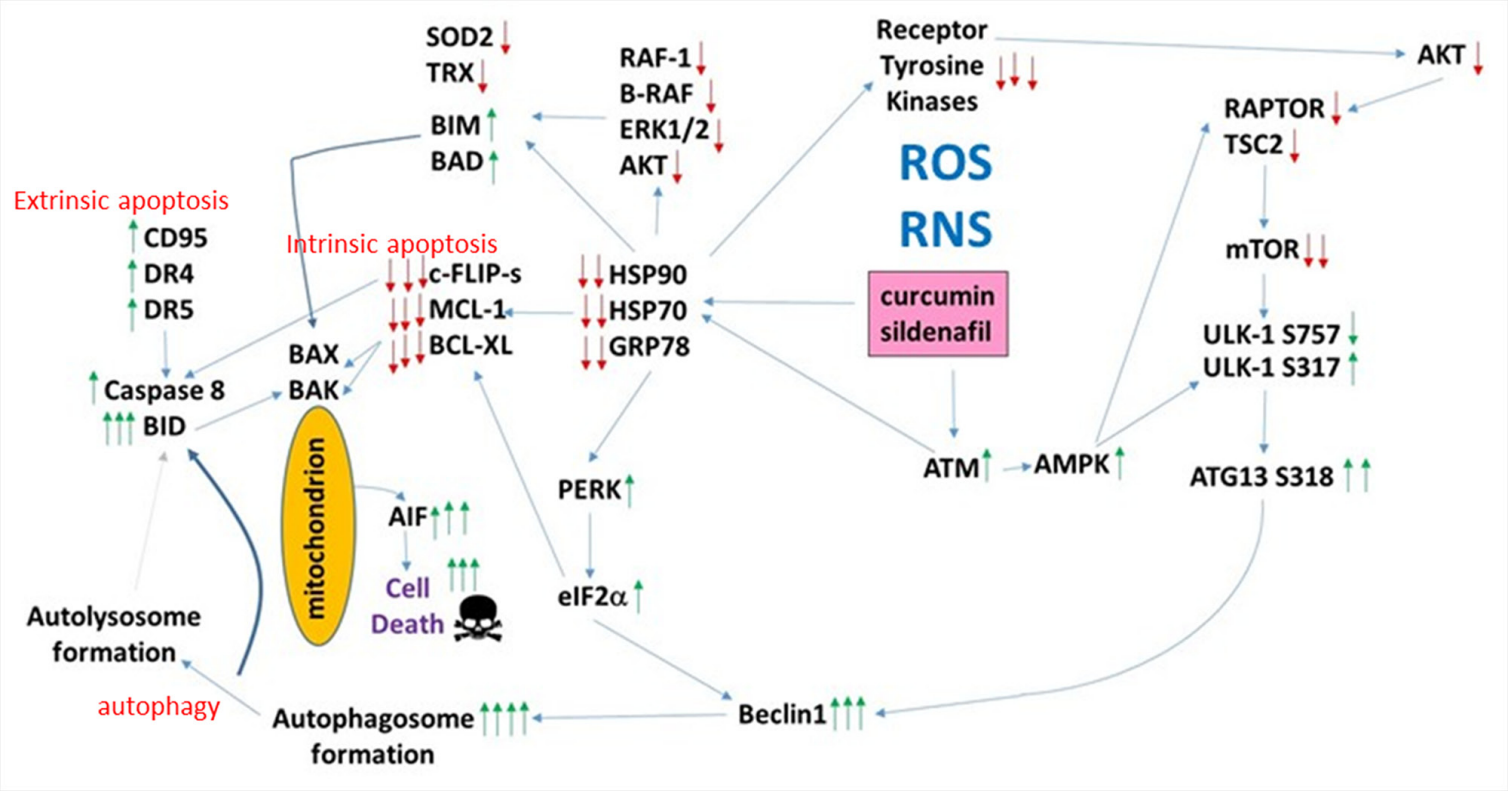
The molecular pathways by which curcumin and sildenafil interact to kill tumor cells Exposure to [curcumin + sildenafil] increases the levels of ROS and RNS in tumor cells which plays a role in mediating DNA damage and reduced expression of multiple chaperones. This, in turn, leads to inactivation of cyto-protective signaling pathways and downstream to reduced expression of protective proteins such as MCL-1, BCL-XL, SOD2 and TRX. GRP78 down-regulation permits ER stress signaling to reduce transcription of protective proteins and to stimulate production of Beclin1. DNA damage signaling from ATM through the AMPK promotes inactivation of mTOR and activation of ULK-1 which through ATG13 stimulates autophagosome production – the elevated Beclin1 levels interact with this signal to produce autophagic flux and autolysosome production. ROS/RNS production facilitates CD95 activation that in coordination with cathepsin B from the lysosomes causes BID cleavage, mitochondrial dysfunction and cell death executed by AIF.

In multiple other studies using PDE5 inhibitors to enhance the anti-tumor effects of various chemotherapies we have demonstrated that death receptor signaling plays a key mechanistic role in the killing process. Knock down of CD95/FADD suppressed [curcumin + sildenafil] lethality as did over-expression of c-FLIP-s, all, again, arguing that sildenafil utilizes the extrinsic pathway to kill tumor cells (Figure 12). Curcumin lethality using an order of magnitude greater concentrations than herein has generally been linked to high levels of ROS and killing through mechanisms downstream of mitochondrial dysfunction: caspases 9/3 and AIF. In addition to death receptor activation and mitochondrial dysfunction, the drug combination induced an ER stress signal via eIF2α that was in part responsible enhanced autophagosome and autolysosome formation. Inappropriate breakdown of lysosomes leads to the release of activated proteases into the cytosol, and we found that knock down of one such protease, cathepsin B, also was one component in drug combination lethality. Overall, our data argue for a highly complex mechanism of cell killing by the combination of curcumin and sildenafil, and clearly many more detailed mechanistic studies will be required to fully define all of the induced cell death processes.

One well known mechanism of high non-physiologic curcumin toxicity towards tumor cells is the generation of reactive oxygen and reactive nitrogen species. Sildenafil in tumor cells can also increase the levels of ROS and NOS, as we recently published [45]. Curcumin and sildenafil interacted in a greater than additive fashion to generate ROS in a prolonged fashion. Furthermore, through eIF2α signaling, which reduces the transcription of many genes, the expression levels of enzymes that would de-toxify the ROS, SOD2 and TRX, were reduced. Knock down of TRX or SOD2 enhanced drug combination killing whereas over-expression of the enzymes suppressed cell death. Thus a key component of the lethal interaction of sildenafil and curcumin occurs via high lethal levels of ROS and RNS.

One mode of action of nitric oxide signaling in cells can be to promote activation of cytoplasmic ATM, normally thought to be in the nucleus and associated with the cellular response to DNA damage. The combined effect of [curcumin + sildenafil] exposure was to activate ATM, an effect that was reduced by knock down of nitric oxide synthase enzymes. In addition, there is a previously described signaling pathway from ATM to phosphorylation of the AMPK on threonine 172, and we found that T172 phosphorylation as well as phosphorylation of the AMPK target ULK-1 S317 were increased in an ATM- and NOS-dependent fashion. Thus nitric oxide signaling through ATM-AMPK-ULK-1 acts to facilitate the toxic autophagosome formation response in curcumin and sildenafil. The precise molecular mechanisms by which nitric oxide acts to cause ATM activation, e.g. inhibition of protein phosphatases; inhibition of chaperone proteins, will require investigation beyond the scope of the present manuscript.

Endoplasmic reticulum stress signaling is mediated through three self-regulatory pathways: through eIF2α, IRE1, and ATF6. Signaling by eIF2α reduces transcription from many cyto-protective genes resulting in the rapid reduction of protein expression for those with short half-lives, e.g. MCL-1, but also increases the activity of others such as NRF2 which feeds forward onto the system by activation of antioxidant genes shutting off reactive oxygen species signaling. IRE1/XBP-1 signaling can increase the levels of chaperones such as GRP78 which promote protein folding and also act to shut of the stress signaling pathways. Signaling by IRE1 and by ATF6 can activate NFκB signaling which most often is considered a cyto-protective signal, though in some systems is a downstream cyto-toxic effector [46]. ATF6 signaling can increase the levels of XBP-1 that collectively also leads to production of cyto-protective chaperones and antioxidant enzymes. However, ATF6 can also signal towards cell death, as observed in our system, where together with ATF4, downstream of PERK, it can act to increase the levels of CHOP thereby stimulating cell death processes. It has been shown by others that nitric oxide -induced apoptosis in macrophages requires ATF4-/ATF6- induced CHOP expression [47].

We have previously published that celecoxib and sildenafil interact *in vitro* and *in vivo* to kill tumor cells. In the present studies curcumin and sildenafil were also shown to interact to suppress tumor growth, an effect further enhanced by co-administration of celecoxib. From multiplex analyses of tumors previously treated with [curcumin + sildenafil] and [curcumin + sildenafil + celecoxib] we discovered that the serum concentrations of M-CSF, CXCL-9, PDGF and G-CSF were increased in response to [curcumin + sildenafil]. The impact of the immune response in any malignancy is still relatively poorly understood. While immune cells can destroy transformed cells, the targeting and accumulation of monocytes and macrophages at tumor sites may actually promote tumor growth and metastases. The growth factor, macrophage colony stimulating factor (M-CSF), is important in promoting monocyte survival and may lead to promotion of tumor growth. It can cooperate with G-CSF in this process [48]. Indeed, it has been suggested that animals deficient in M-CSF had fewer tumor metastases than animals producing normal levels of M-CSF in an animal model of breast cancer [49]. The promotion of metastasis is thought to result from the potential of M-CSF to recruit mononuclear phagocytes, increase VEGF levels [50], and enhance angiogenesis in an ERK- and Sp1-dependent mechanism [51]. However, in the current study multiplex analysis indicated that serum concentration of VEGF was unaltered in response [curcumin + sildenafil]. It may prove interesting to investigate the potential of [curcumin + sildenafil] in suppressing VEGF secretion and expression.

The serum concentration of CXCL1 was significantly reduced in response to [curcumin + sildenafil] treatment. Reduced CXCL1 levels have been shown to result in decreased colorectal tumor growth and to suppress tumorigenic growth of K-RAS mutant colon cancer cells [52]. Furthermore, high CXCL1 expression is known to be a poor prognostic biomarker in metastatic colon cancer [53]. Cancer cells that overexpress CXCL1 and 2 by transcriptional hyper-activation or 4q21 amplification are primed for survival in metastatic sites and increased anchorage-independent growth of murine fibroblasts and human colon cancer cells [54]. We discovered that the plasma concentration of TNFα was not altered by any treatment. Chemotherapeutic agents can trigger a stromal reaction within the tumor leading to TNF-α production by endothelial and other stromal cells. TNF-α can heighten the expression of CXCL1/2 in cancer cells, thus amplifying the CXCL1/2-S100A8/9 loop and causing chemo-resistance [54]. Tumor expression of CCL5 was reduced following [curcumin + sildenafil] treatment. CCL5 has been shown to contribute to signaling via CCR1, CCR3 and CCR5 [55]. The CCR1–CCL5 axis which has been linked to malignant progression of hepatocellular carcinoma and by the CCR5-CCL5 axis in colon cancer [56]. Collectively the reduced levels of CXCL-1 and CCL5 argue that the direct and indirect stimulation of tumor cell growth by immunological factors and the immune system is reduced in tumors previously exposed to [curcumin + sildenafil].

The most notable difference in the cytokine profiles between plasma from [curcumin + sildenafil] treated animals and [curcumin + sildenafil + celecoxib] treated animals was the profound increase in the plasma levels of IL-12 (p40). An increase in IL-12 expression has been shown to have a potent anti-tumor activity and may increase the potential for activation of an anti-tumor host immune response. IL-12 will increase the production of IFN-γ, from natural killer (NK) and T cells [57], in addition to increasing the growth and cytotoxicity of activated NK cells, CD8^+^ and CD4^+^ T cells [58] and shifting differentiation of CD4^+^ Th0 cells toward the Th1 phenotype [59]; and enhancement of antibody-dependent cellular cytotoxicity against tumor cells [60]. Furthermore, IL-12 may exert a potent anti-angiogenic effects via induction of anti-angiogenic cytokine and chemokine production [61]; and changes in processing and increasing expression of MHC class I molecules [62].

PDGF isoforms and PDGF receptors have important functions in the regulation of growth and survival of certain cell types during embryonal development and e.g. tissue repair in the adult. Over-activity of PDGF receptor signaling, by over receptor / ligand expression or receptor mutational events, may drive tumor cell growth [63]. Studies in colorectal cancer patients have suggested that PDGF over-expression correlates with vascular invasion, where invasion was significantly greater in patients expressing PDGF-BB at a high level than in those at a low level [64]. Patients with high PDGF-BB expression also had a significantly poorer survival rate than those with low PDGF-BB expression [65]. In theory, to develop our concept further towards the clinic and to counteract this increase in PDGF-BB induced by [curcumin + sildenafil], a number of FDA-approved inhibitors of PDGFRα/β kinases are available, including regorafenib, imatinib, sunitinib, sorafenib, pazopanib and nilotinib. None of these inhibitors are precisely specific for PDGFRα/β; they all have overlapping profiles of inhibition of other kinases, including chaperone proteins. Thus, imatinib, in addition to inhibiting PDGFRα/β, inhibits the stem cell receptor (KIT) and ABL kinases, and sunitinib inhibits vascular endothelial cell growth factor (VEGF) receptors and FLT3; sorafenib and regorafenib have an inhibitory profile similar to sunitinib, but they also inhibit the serine/threonine kinases RAF-1 and B-RAF, as well as HSP90, HSP70 and GRP78. Our present findings argued that regorafenib could enhance [curcumin + sildenafil] lethality in a tumor cell population that had previously been exposed to the drug combination. Although the lack of precise specificity can contribute to side effects in patients and can be seen as a disadvantage, experience from our laboratory has shown that it is often advantageous to inhibit more than one receptor tyrosine kinase in any tumor treatment. Collectively, our prior findings with [celecoxib + sildenafil], and our present data with [curcumin + sildenafil] and [curcumin + sildenafil + celecoxib] strongly support the proposal of clinical studies in advanced colon cancer patients with metastatic liver disease combining the standard of care agent regorafenib with [curcumin + sildenafil + celecoxib].

## MATERIALS AND METHODS

### Materials

Sildenafil and celecoxib were purchased from Selleckchem (Houston, TX). Curcumin was purchased from Sigma-Aldrich (St. Louis, MO). Trypsin-EDTA, DMEM, RPMI, penicillin-streptomycin were purchased from GIBCOBRL (GIBCOBRL Life Technologies, Grand Island, NY). Tumor cell lines were purchased from the ATCC and were not further validated beyond that claimed by ATCC. Cells were re-purchased every ∼6 months. The plasmid to express GRP78/BiP/HSPA5 was kindly provided to the Dent Laboratory at VCU (the laboratory where the current studies were performed) and by Dr. A.S. Lee (University of Southern California, Los Angeles, CA); all other plasmids were purchased from Adgene, (Cambridge, MA). Commercially available validated short hairpin RNA molecules to knock down RNA / protein levels were from Qiagen (Valencia, CA). Supplementary Figure 13 presents control immunofluorescence studies demonstrating the knock down of individual proteins by each siRNA.

### Methods

#### Culture and *in vitro* exposure of cells to drugs

All cell lines were cultured at 37 °C (5% (v/v CO_2_) *in vitro* using DMEM supplemented with dialyzed 5% (v/v) fetal calf serum and 10% (v/v). For short term cell killing assays, immunoblotting studies, cells were plated at a density of 3 x 10^3^ per cm^2^ (∼2 x 10^5^ cells per well of a 12 well plate) and 48h after plating treated with various drugs, as indicated. *In vitro* drug treatments were generally from a 100 mM stock solution of each drug and the maximal concentration of Vehicle carrier (VEH; DMSO) in media was 0.02% (v/v). All drug stock solutions were stored at -80 ^0^C. Cells were not cultured in reduced serum media during any study in this manuscript.

#### Antibodies used

Antibodies to BAX, BAK, BCL-XL, CHOP, c-FLIP, IRE1, RIP1, iNOS, FADD, Cathepisin B, mTOR, phospho-mTOR S2448 and S2481, phospho-RAPTOR S792, TSC2 T1426, PTEN, phospho-PTEN S380, ATF6, eNOS, AIF, XBP1, NOXA, PUMA, ATG5 phospho-ATG13 S318, Beclin-1, AKT, phospho-AKT T308, eiF2α, phospho-eiF2α S51, phospho p65 S536, ATF4, PGKI and II, TRX, SOD, ATM, phospho-ATM S1981, AMPKα, phospho-AMPK T172, phospho-ULK1 S757, S317, STAT3, STAT5, p70 S6K, phospho-ERK1/2, GRP78, HSP70 and HSP90, phospho-γH2AX, were purchased from Cell Signaling Technology, (Danvers, MA). PERK, CD95 and caspase 9 antibodies, were purchased from Santa Cruz Biotechnology, (Dallas, TX).

#### Transfection of cells with siRNA or with plasmids

For Plasmids: Cells were transfected 24h after plating. Plasmids expressing a specific mRNA (or siRNA) or appropriate vector control plasmid DNA was diluted in 50μl serum-free and antibiotic-free medium (1 portion for each sample). Concurrently, 2μl Lipofectamine 2000 (Invitrogen), was diluted into 50μl of serum-free and antibiotic-free medium (1 portion for each sample). Diluted DNA was added to the diluted Lipofectamine 2000 for each sample and incubated at room temperature for 30 min. This mixture was added to each well / dish of cells containing 200μl serum-free and antibiotic-free medium for a total volume of 300 μl, and the cells were incubated for 4 h at 37 °C. An equal volume of 2x medium was then added to each well. Cells were incubated for 24h, then treated with drugs.

Transfection for siRNA: Cells from a fresh culture growing in log phase as described above, were transfected 24h after plating. Prior to transfection, the medium was aspirated and serum-free medium was added to each plate. For transfection, 10 nM of the annealed siRNA, the positive sense control doubled stranded siRNA targeting GAPDH or the negative control (a “scrambled” sequence with no significant homology to any known gene sequences from mouse, rat or human cell lines) were used. Ten nM siRNA (scrambled or experimental) was diluted in serum-free media. Four μl Hiperfect (Qiagen) was added to this mixture and the solution was mixed by pipetting up and down several times. This solution was incubated at room temp for 10 min, then added drop-wise to each dish. The medium in each dish was swirled gently to mix, then incubated at 37 °C for 2h. Serum-containing medium was added to each plate, and cells were incubated at 37^°^C for 24h before then treated with drugs (0-24h). Additional immuno-fluorescence / live-dead analyses were performed at the indicated time points. Supplementary Figure 13 shows immuno-fluorescent imaging of a range of cell proteins following specific siRNA knockdown / or the use of a “scrambled” sequence with no significant homology to any known gene sequences from mouse, rat or human cell lines.

#### Animal Studies

Studies were performed according to USDA regulations under VCU IACUC protocol AD20008. Immuno-competent BALB/c mice (∼20 g) were injected with 1 x 10^6^ CT26 cells into their rear flank (10 animals per treatment group +/- SEM). Tumors were permitted to form for 7 days with tumors at that time exhibiting a mean volume of ∼45 mm^3^. Mice were treated by oral gavage once every day BID for five days as indicated in the Figure and Figure Legend with vehicle (Cremophore, Sigma-Aldrich, St Louis MO, 63013, USA); with sildenafil (10 mg/kg) on days 1-5; with celecoxib (10 mg/kg) on Days 1-5; or with curcumin (50 mg/kg) on Days 1-5, or in combination as indicated. After cessation of drug treatment tumors are again calipered as indicated in the Figure and tumor volume was assessed up to 20-30 days later.

### Detection of cell death by trypan blue

Typan blue exclusion was used to assess cell viability at each experimental time point. Floating cells were isolated along with attached cells that were harvested by trypsinization with Trypsin/EDTA for ∼3 min at 37°C. Following isolation, the total cell population for each experimental point was assessed for cell viability.

### Detection of protein expression and protein phosphorylation by immuno-fluorescence using a Hermes WiScan machine

Cells (4 x 10^3^) were plated into each well of a 96 well plate and allowed to grow over night. Depending upon the specific experiment, cells were then either genetically manipulated, or treated with drugs. For genetic manipulation, cells were transfected with plasmids or siRNA molecules and incubated for an additional 24h. Cells were treated with vehicle control or with drugs at the indicated final concentrations, alone or in combination. Cells were isolated for processing at various times following drug exposure. For immuno-fluorescence studies, after centrifugation, cell growth media was removed and cells were fixed in place in 0.4% paraformaldehyde for 10 minutes at room temperature. The cells were then permeabilized using ice cold PBS containing 0.5% Triton X-100. After 30 min the cells were washed three times with ice cold PBS and pre-blocked with rat serum for 3 hours. Following pre-blocking, cells were incubated with a primary antibody for the detection and expression / phosphorylation of a given protein (usually at 1:100 dilution from a commercial vendor) overnight at 37°C. Following overnight incubation, cells were washed three times with PBS followed by incubation with a secondary antibody containing an associated fluorescent red or green chemical tag, for 3 hours. Following this incubation, the cells were washed three times in PBS and 100 μl of PBS was added to each well for assessment of protein expression. The cells were visualized at either 10X or 60X in the Hermes machine. All immunofluorescent images for each individual protein / phospho-protein were recorded using the standardized settings to ensure that signal level for each image was directly comparable to signal level in the control and drug treated cells. Similarly, for presentation, the enhancement of image brightness/contrast using PhotoShop CS6 was simultaneously performed for each individual set of protein/phospho-protein to permit direct comparison of the image intensity between treatments. All immune-fluorescent images were initially visualized at 75 dpi using an Odyssey infrared imager (Li-Cor, Lincoln, NE), then processed at 9999 dpi using Adobe Photoshop CS6. For presentation, immunoblots were digitally assessed using the provided Odyssey imager software. Images have their color removed and labeled figures generated in Microsoft PowerPoint.

### Assessment of autophagy

Cells were transfected with a plasmid to express a green fluorescent protein (GFP) and red fluorescent protein (RFP) tagged form of LC3 (ATG8). For analysis of cells transfected with the GFP-RFP-LC3 construct, the GFP/RFP-positive vesicularized cells were examined under the ×40 objective of a Zeiss Axiovert fluorescent microscope. At each time point the mean number of intense GFP/RFP-LC3 punctae per cell was determined from at least 40 cells per condition.

### Multiplex assays

Using a MAGPIX multiplex instrument with associated software (Bio-Rad, CA), the following Bio-Plex assay plates were used in our assays of mouse plasma cytokines: bio-plex-pro-mouse-cancer-biomarker-panel-1-23-plex; bio-plex-pro-mouse-cytokine-standard 9 plex; bio-plex-pro-mouse-single multiplex phospho target-pAKT S308d; bio-plex-pro-mouse-single multiplex phospho target-pmTOR S2448.

### Analysis of ROS levels

ROS levels were determined in a vector 3 plate reader (PerkinElmer Life and Analytical Sciences). In brief, cancer cells were plated in 96-well plates. Cells were loaded for 30 min with either dihydro-DCF (10 μM) which is sensitive to oxidation by hydroxyl radicals and peroxynitrite directly and hydrogen peroxide; or 3-amino,4-aminomethyl-2′,7′-difluorescein (DAF-FM DA, 4 μM) which is sensitive to oxidation by NO. Cells were treated with drugs and fluorescence measured 2 and 6 h afterwards. Data are presented corrected for basal fluorescence of vehicle-treated cells at each time point.

### Data analysis

Comparison of the effects of various treatments was performed using one-way analysis of variance and a two tailed Student’s *t*-test. Statistical examination of *in vivo* animal survival data utilized log rank statistical analyses between the different treatment groups. Differences with a *p*-value of < 0.05 were considered statistically significant. Experiments shown are the means of multiple individual points from multiple experiments (± SEM).

## SUPPLEMENTARY MATERIALS FIGURES



## Abbreviations

ERK: extracellular regulated kinase
PI3K: phosphatidyl inositol 3 kinase
ca: constitutively active
dn: dominant negative
ER: endoplasmic reticulum
mTOR: mammalian target of rapamycin
JAK: Janus Kinase
STAT: Signal Transducers and Activators of Transcription
MAPK: mitogen activated protein kinase
PTEN: phosphatase and tensin homologue on chromosome ten
ROS: reactive oxygen species
CMV: empty vector plasmid or virus
si: small interfering
SCR: scrambled
IP: immunoprecipitation
VEH: vehicle
Cur: curcumin
Sil: sildenafil
Cel: celecoxib
