# Supplementary material for: Curcumin interacts with sildenafil to kill GI tumor cells via endoplasmic reticulum stress and reactive oxygen/ nitrogen species
Source: Oncotarget. 2017 Aug 2;8(59):99451–69. doi: 10.18632/oncotarget.19807 (PMC5725106; doi:10.18632/oncotarget.19807)
Supplement: Supplementary file 1 [file oncotarget-08-99451-s001.pdf]

Curcumin interacts with sildenafil to kill GI tumor cells via endoplasmic reticulum stress and reactive oxygen/ nitrogen species

SUPPLEMENTARY MATERIALS

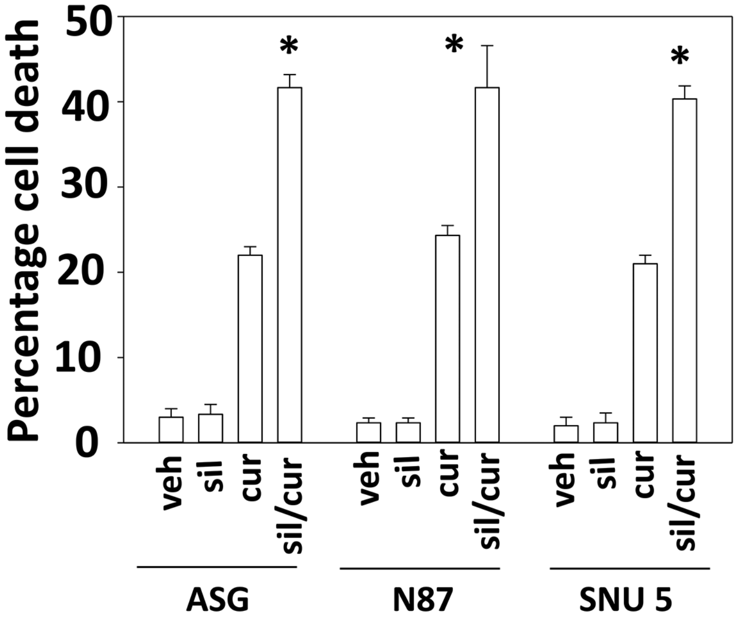

Supplementary Figure 1: Curcumin interacts with sildenafil and with celecoxib to kill stomach tumor cells - Stomach cancer cells were treated with vehicle control, curcumin (2.0 µM), sildenafil (2.0 µM), celecoxib (2.0 µM) or the drugs in the indicated combinations for 24h. Cell death was measured by trypan blue exclusion (n = 3 +/-SEM) * $p < 0.05$ greater than individual drug treatments.

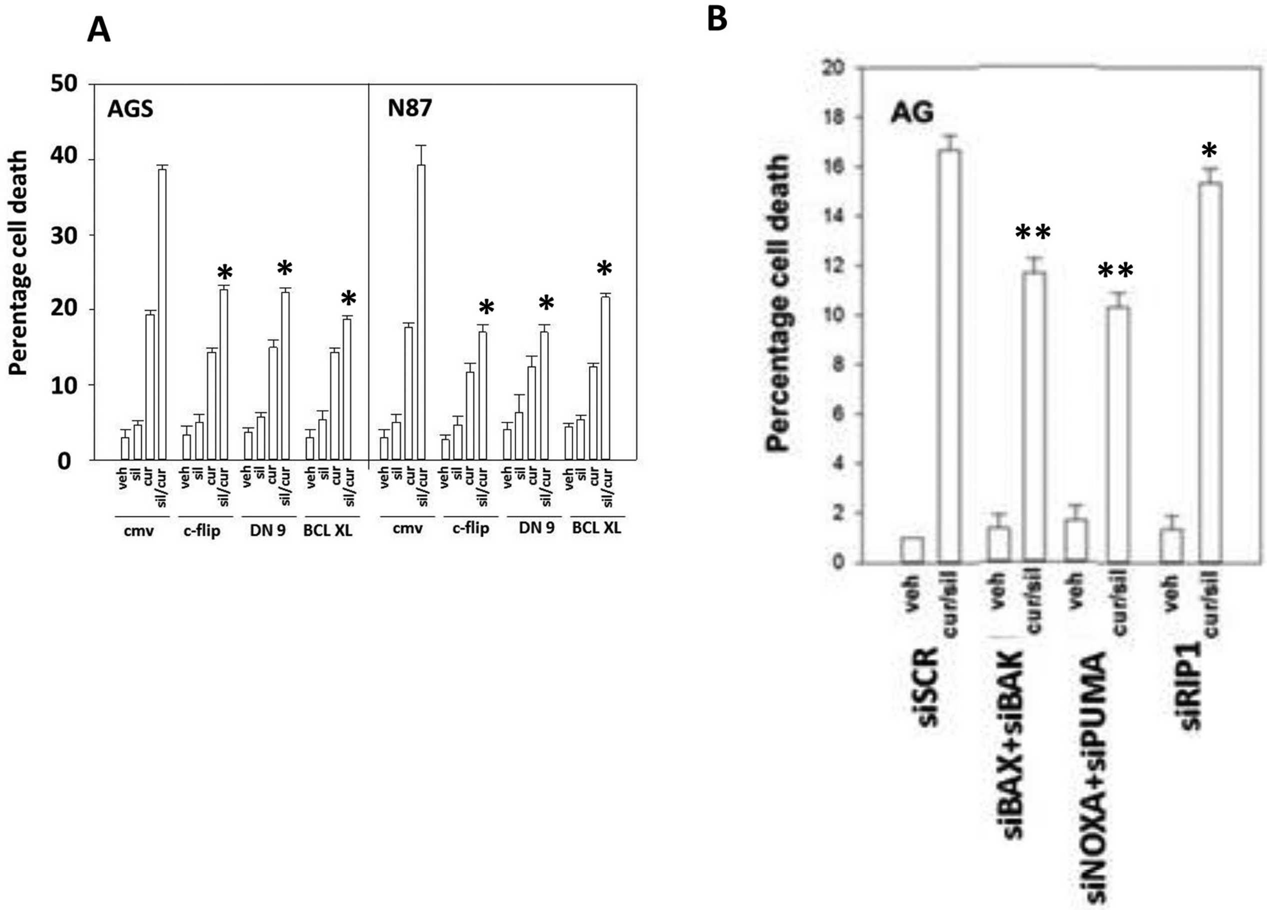

Supplementary Figure 2: Curcumin and sildenafil kill through activation of the extrinsic and intrinsic pathways - Twenty-four h after plating cells were treated for 24h with vehicle, curcumin (2.0 µM), sildenafil (2.0 µM) or the drugs in combination. Cell death was measured by trypan blue exclusion (n = 3 +/-SEM) * $p < 0.05$ less killing than corresponding value in CMV transfected. **(A)** Stomach cells were transfected with empty vector control or to express: c-FLIP-s; BCL-XL; or dominant negative caspase 9. **(B)** Stomach cells were transfected with a scrambled siRNA control (siSCR) or with siRNA molecules to knock down: BAX+BAK; NOXA+PUMA; and RIP1. (n = 3 +/-SEM) * $p < 0.05$ less killing than corresponding value in siSCR transfected; ** $p < 0.05$ less killing than corresponding value in siRIP-1 transfected.

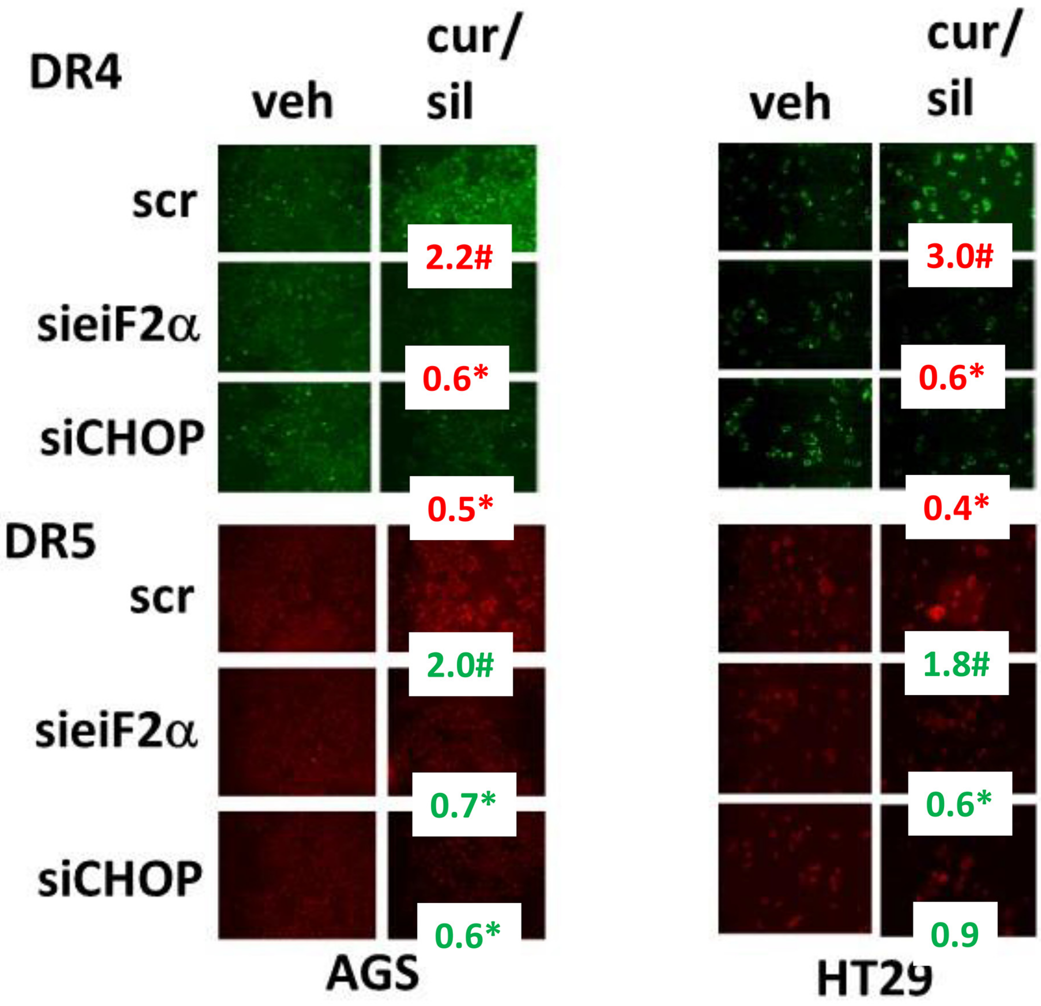

Supplementary Figure 3: Signaling by eIF2α-CHOP promotes the expression of the death receptors DR4 and DR5. Tumor cells were transfected with a scrambled siRNA (scr) or with siRNA molecules to knock down the expression of eiF2α or of CHOP. Twenty-four h after transfection cells were treated with vehicle control or with [curcumin (2.0 μM) + sildenafil (2.0 μM)] for 6h. Cells were fixed in place and the total cellular expression of DR4 and DR5 determined by immunofluorescence. (n = 3 +/-SEM) * $p < 0.05$ less than corresponding value in vehicle control; $^{\#}$ $p < 0.05$ greater than corresponding value in vehicle control.

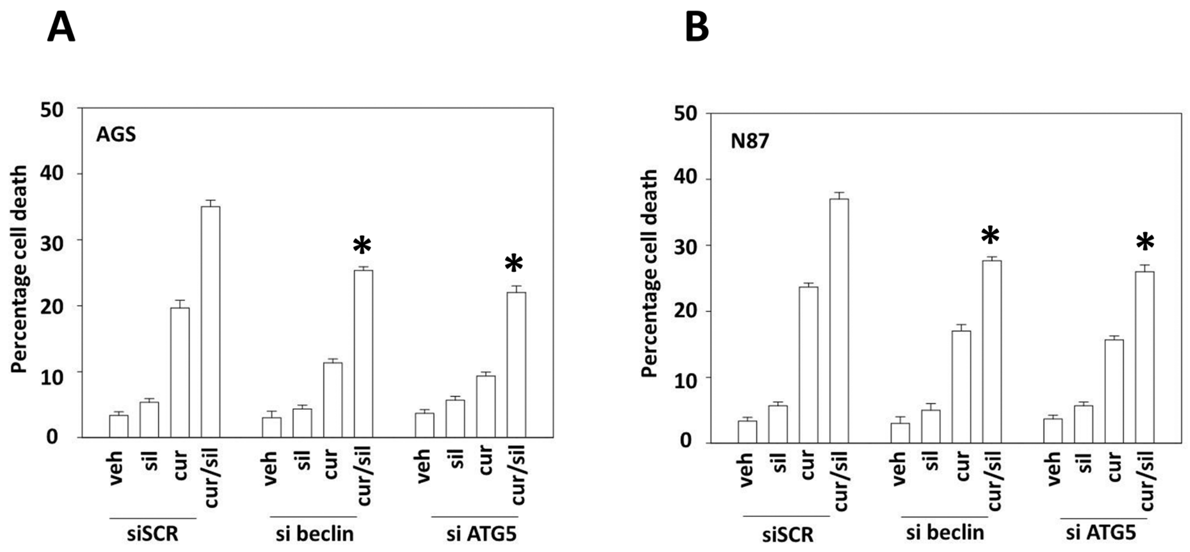

Supplementary Figure 4: Modulation of autophagy can suppress or enhance [curcumin + sildenafil] lethality. AGS stomach cancer cells **(A)** and N87 stomach cancer cells **(B)**, were transfected with a scrambled siRNA control (siSCR) or with siRNA molecules to knock down: ATG5 or Beclin1. 24h later, cells were treated with vehicle control (0.01% DMSO), curcumin (2.0 µM), sildenafil (2.0 µM), or the drugs in combination for 24h. Cell death was measured by trypan blue exclusion (n = 3 +/-SEM) * $p < 0.05$ less than individual drug treatments in siSCR treated cells.

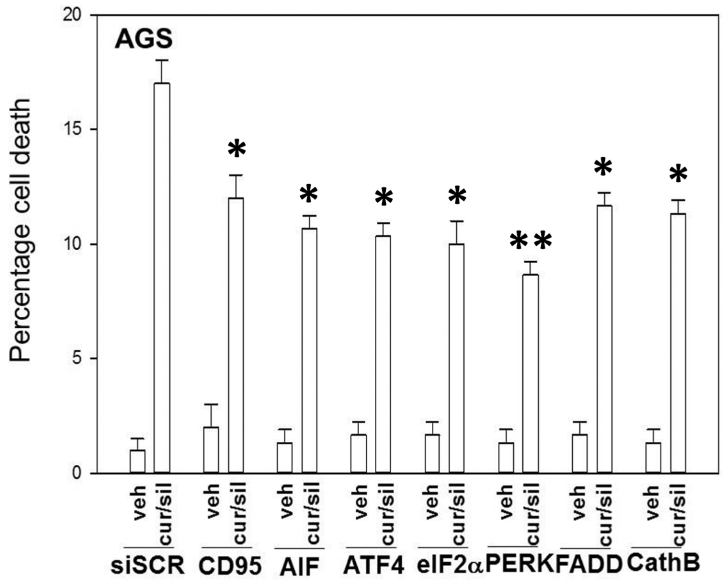

Supplementary Figure 5: Endoplasmic reticulum stress signaling induced by [curcumin + sildenafil] through PERK-eIF2α causes tumor cell death. Stomach tumor cells were transfected with a scrambled siRNA control or with siRNA molecules to knock down the expression of: CD95; AIF; ATF4; eIF2α; PERK; FADD; and cathepsin B. Twenty-four h after transfection cells were treated with vehicle control or with [curcumin (2.0 µM) + sildenafil (2.0 µM)] for 12h. Cell death was measured by trypan blue exclusion (n = 3 +/-SEM). * $p < 0.05$ less than corresponding value in siSCR cells; ** $p < 0.01$ less than corresponding value in siSCR cells.

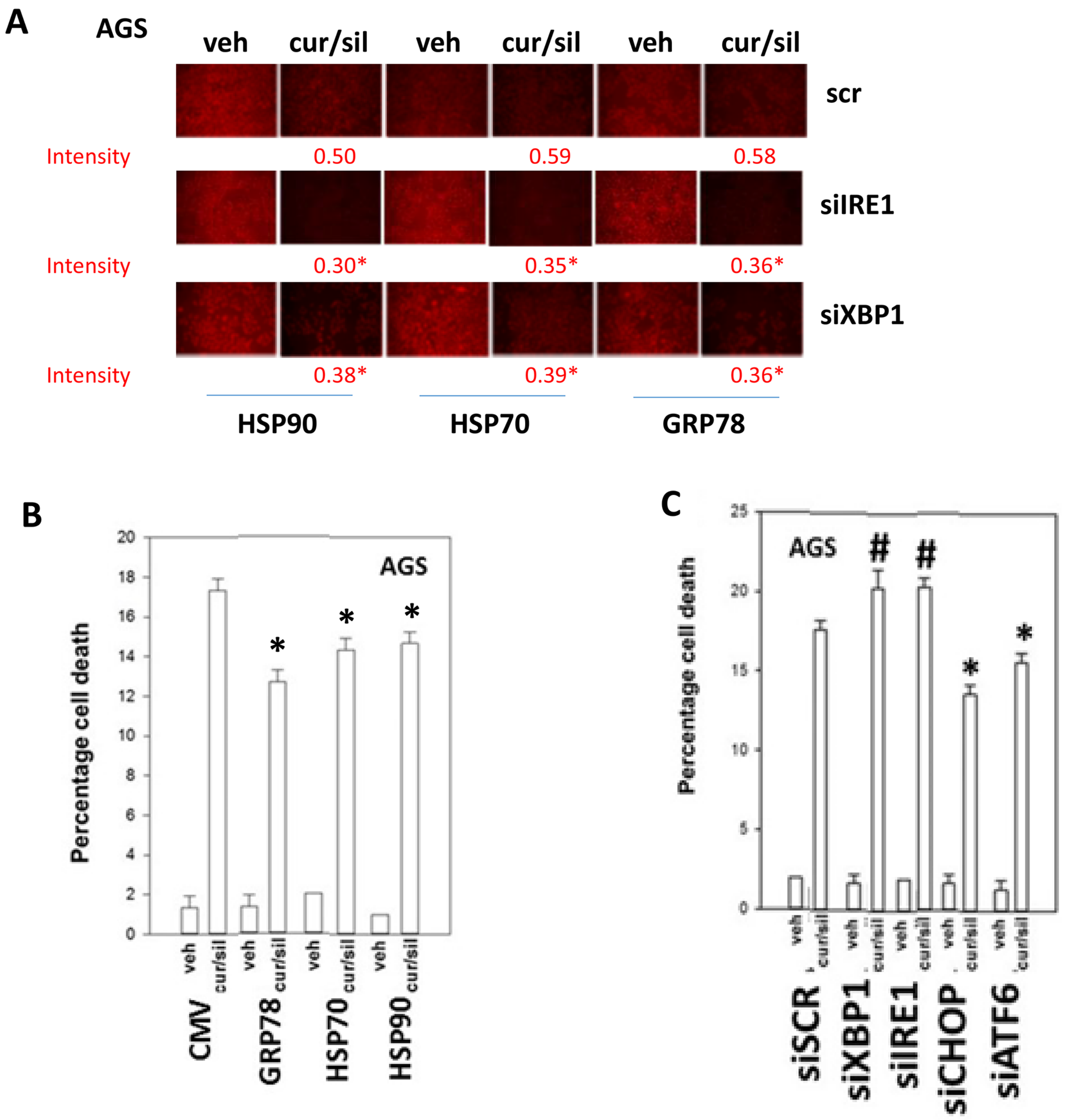

Supplementary Figure 6: PERK- and ATF6- dependent signaling facilitates [curcumin + sildenafil] toxicity whereas IRE1/XBP1 signaling via chaperone expression is protective. Stomach tumor cells were transfected with a scrambled siRNA control or with siRNA molecules to knock down the expression of: XBP1 or IRE1. Twenty-four h after transfection cells were treated with vehicle control or with [curcumin (2 μM) + sildenafil (2 μM) for a further 6h. Cells were fixed in situ and immuno- fluorescence at 10X performed to determine the expression levels of HSP90, HSP70, GRP78. (n = 3 +/- SEM) * p < 0.05 less than corresponding value in siSCR cells. **(B)** Tumor cells were transfected with an empty vector plasmid (CMV) or plasmids to express: GRP78; HSP90; or HSP70. Twenty-four h after transfection cells were treated with vehicle control or with [curcumin (2 μM) + sildenafil (2 μM) for a further 12h. Cell death was measured by trypan blue exclusion (n = 3 +/-SEM). * p < 0.05 less than corresponding value in siSCR cells. **(C)** Stomach tumor cells were transfected with a scrambled siRNA control or with siRNA molecules to knock down the expression of: XBP1; IRE1; CHOP; or ATF6. Twenty-four h after transfection cells were treated with vehicle control or with [curcumin (2 μM) + sildenafil (2 μM) for a further 12h. Cell death was measured by trypan blue exclusion (n = 3 +/- SEM). # p < 0.05 greater than corresponding value in siSCR cells; * p < 0.05 less than corresponding value in siSCR cells.

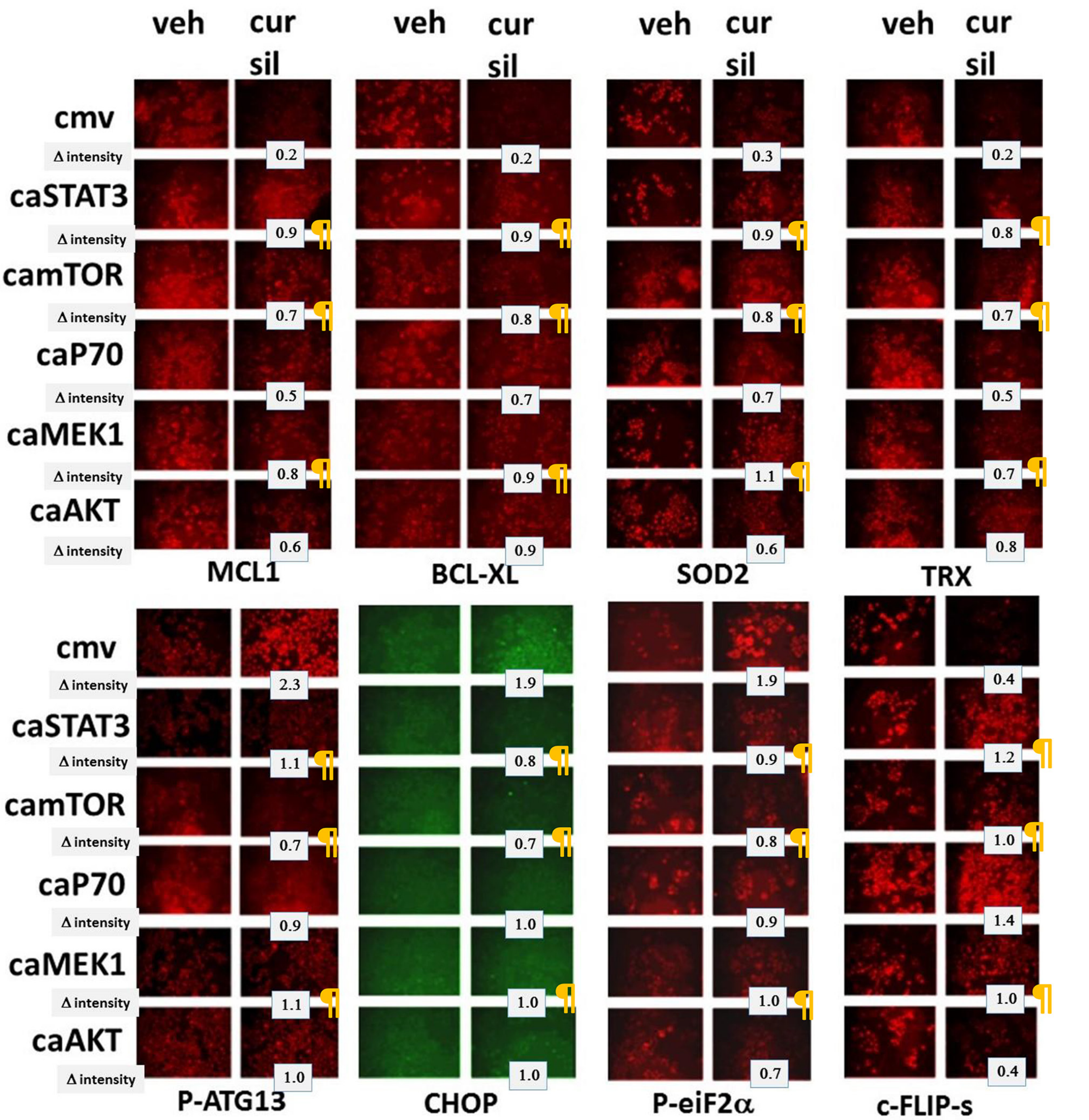

Supplementary Figure 7: Expression of activated STAT3 and to a lesser extent activated mTOR prevents the [curcumin + sildenafil]-induced reductions in MCL-1, BCL-XL, SOD2, TRX and c-FLIP-s expression. Tumor cells were transfected with an empty vector plasmid (CMV) or with plasmids to express: activated STAT3; activated mTOR; activated p70 S6K; activated MEK1; activated AKT. Twenty-four h after transfection cells were treated with vehicle control or with [curcumin (2.0 μM) + sildenafil (2.0 μM)] for 6h. Cells were fixed in place and the total cellular expression of MCL-1, BCL- XL, SOD2, TRX, CHOP and c-FLIP-s determined as well as the phosphorylation of ATG13 S318 and eIF2α S51 determined by immunofluorescence and the fold change in expression / phosphorylation in relation to vehicle control shown (n = 3 +/- SEM). ¶ $p > 0.05$ compared to its corresponding vehicle control value.

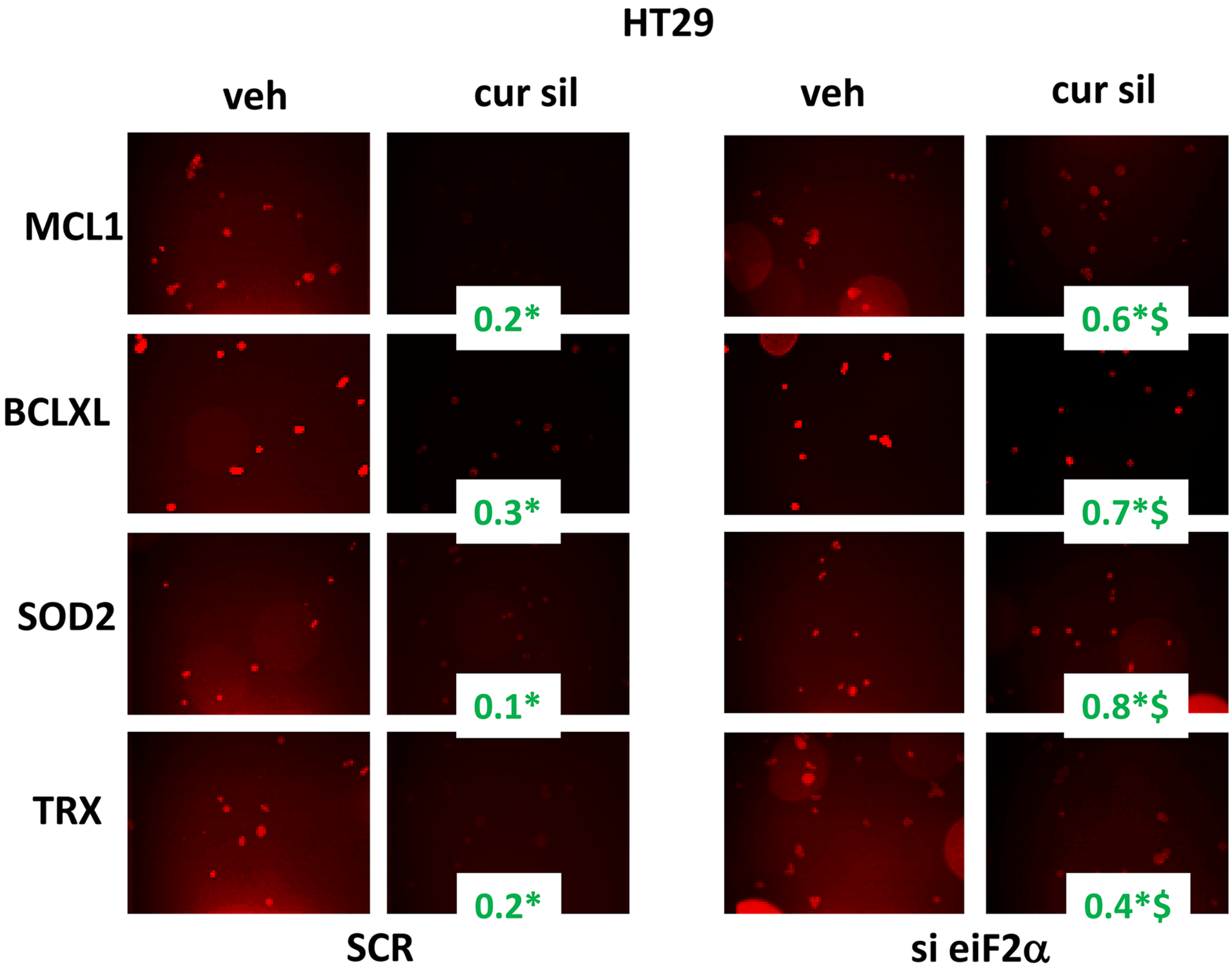

Supplementary Figure 8: Inhibition of eIF2α-dependent stress signaling abolishes the drug-induced decline in the expression of MCL-1, BCL-XL, SOD2 and TRX. Tumor cells were transfected with a scrambled siRNA (scr) or with an siRNA molecule to knock down the expression of eiF2α. Twenty- four h after transfection cells were treated with vehicle control or with [curcumin (2.0 µM) + sildenafil (2.0 µM)] for 6h. Cells were fixed in place and the total cellular expression of MCL-1, BCL-XL, SOD2 and TRX. The fold change in expression / phosphorylation in relation to vehicle control shown (n = 3 +/- SEM). * $p < 0.05$ less than its corresponding vehicle control value; $ $p < 0.05$ greater than its corresponding value in SCR cells.

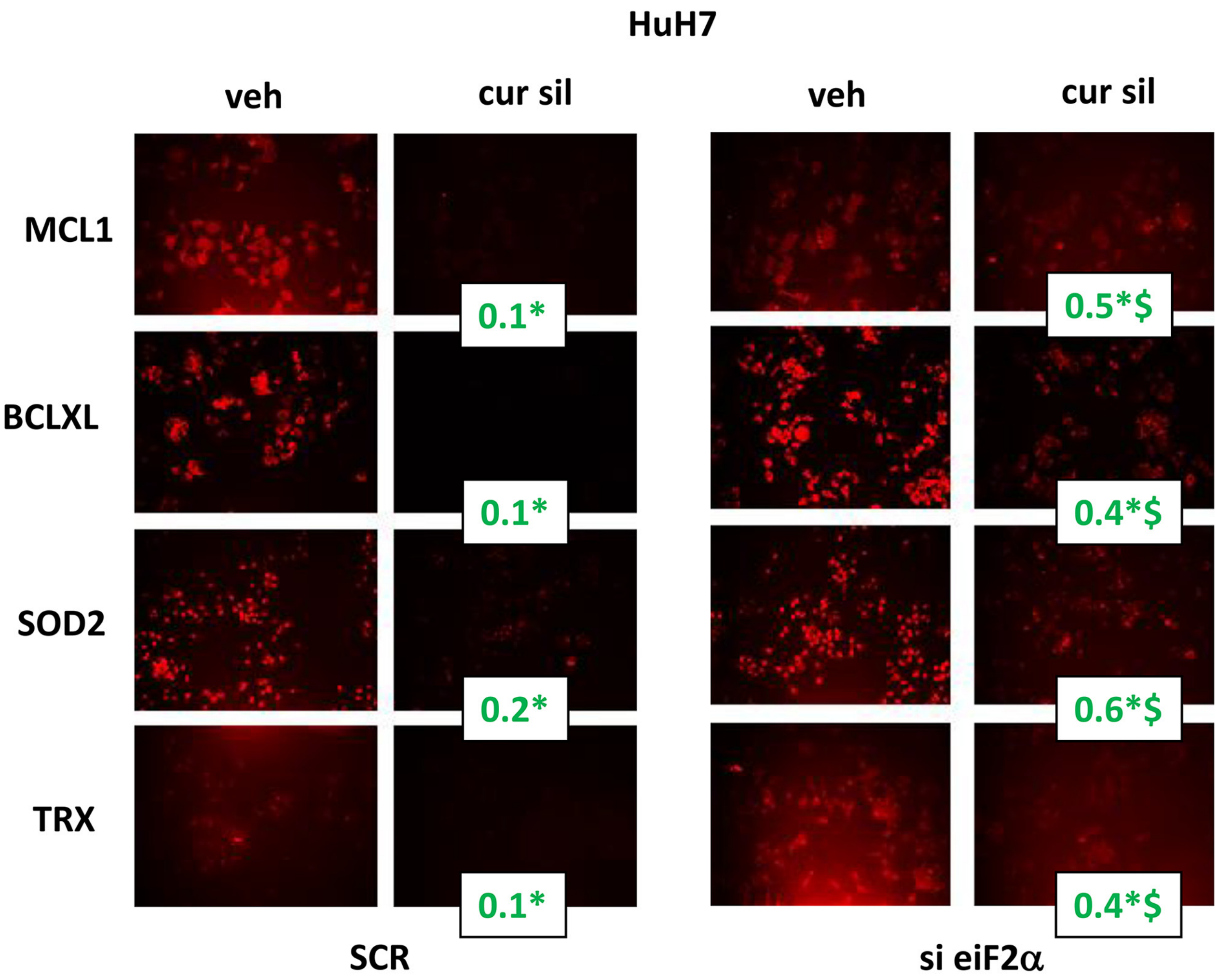

Supplementary Figure 9: Inhibition of eIF2α-dependent stress signaling abolishes the drug-induced decline in the expression of MCL-1, BCL-XL, SOD2 and TRX. HuH7 tumor cells were transfected with a scrambled siRNA (scr) or with an siRNA molecule to knock down the expression of eiF2α. Twenty- four h after transfection cells were treated with vehicle control or with [curcumin (2.0 µM) + sildenafil (2.0 µM)] for 6h. Cells were fixed in place and the total cellular expression of MCL-1, BCL-XL, SOD2 and TRX. The fold change in expression / phosphorylation in relation to vehicle control shown (n = 3 +/- SEM). * $p < 0.05$ less than its corresponding vehicle control value; $ $p < 0.05$ greater than its corresponding value in SCR cells.

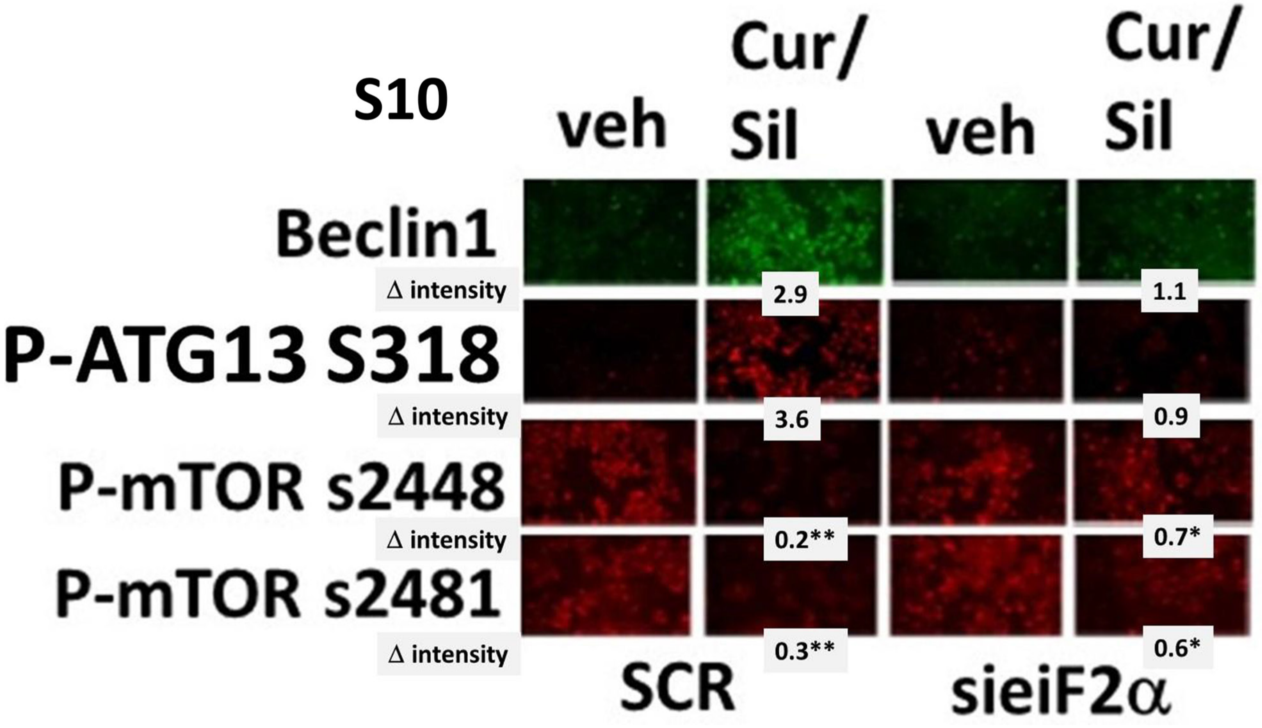

Supplementary Figure 10: Inhibition of eIF2α-dependent stress signaling abolishes the drug-induced expression of Beclin1 and prevents ATG13 S318 phosphorylation. HCT116 tumor cells were transfected with a scrambled siRNA (scr) or with an siRNA molecule to knock down the expression of eiF2α. Twenty-four h after transfection cells were treated with vehicle control or with [curcumin (2.0 μM) + sildenafil (2.0 μM)] for 6h. Cells were fixed in place and the total cellular expression of Beclin1 determined as well as the phosphorylation of ATG13 S318, mTOR S2448 and mTOR S2481 by immunofluorescence. The fold change in expression / phosphorylation in relation to vehicle control shown (n = 3 +/- SEM). * $p < 0.05$ less than its corresponding vehicle control value; ** $p < 0.01$ less than its corresponding vehicle control value.

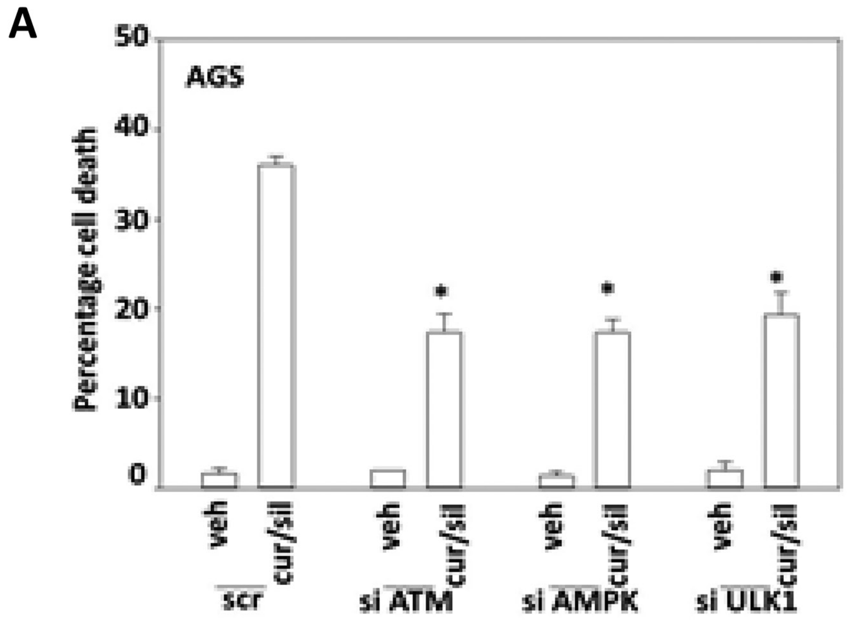

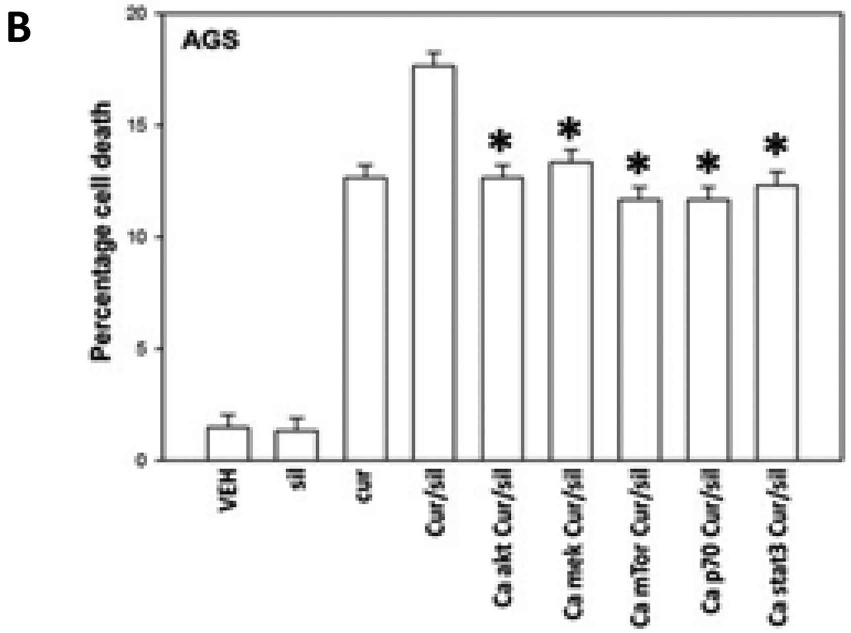

Supplementary Figure 11: [Curcumin + sildenafil] treatment inactivates multiple cyto-protective signaling pathways. **(A)** Stomach tumor cells were transfected with a scrambled siRNA (siSCR) or an siRNA to knock down expression of AMPKα, ATM or ULK-1. Twenty-four h after transfection cells were treated with vehicle control or with [curcumin (2 µM) + sildenafil (2 µM) for a further 24h. Cell viability was determined by trypan blue exclusion (n = 3 +/-SEM) * $p < 0.05$ less than corresponding value in SCR cells. **(B)** Stomach tumor cells were transfected with an empty vector plasmid (CMV) or plasmids to express: activated AKT; activated MEK1; activated mTOR; activated p70 S6K; and activated STAT3. Twenty-four h after transfection cells were treated with vehicle control or with [curcumin (2 µM) + sildenafil (2 µM) for a further 12h. Cell death was measured by trypan blue exclusion (n = 3 +/-SEM). * $p < 0.05$ less than corresponding value in CMV cells.

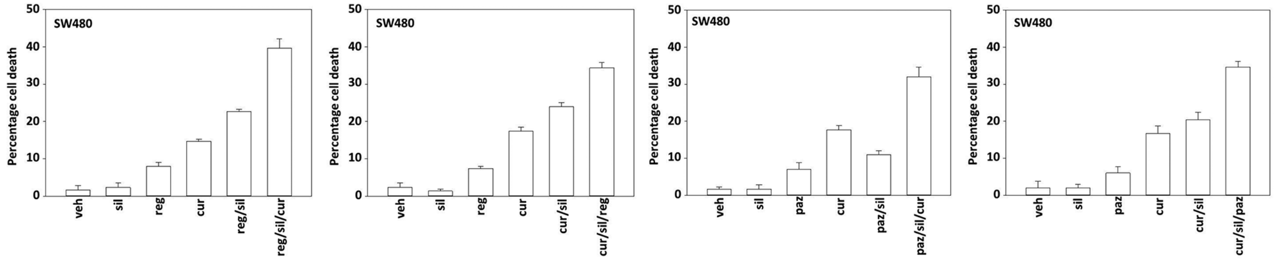

Supplementary Figure 12: Regorafenib and pazopanib both enhance [curcumin + sildenafil] lethality in stomach cells. Stomach cancer cell lines were treated with vehicle control, [curcumin (2.0 µM) + sildenafil (2.0 µM)], regorafenib (1.0 µM), pazopanib (1.0 µM) or the drugs in combination as indicated in each graph for 12h. Cell death was measured by trypan blue exclusion (n = 3 +/-SEM).

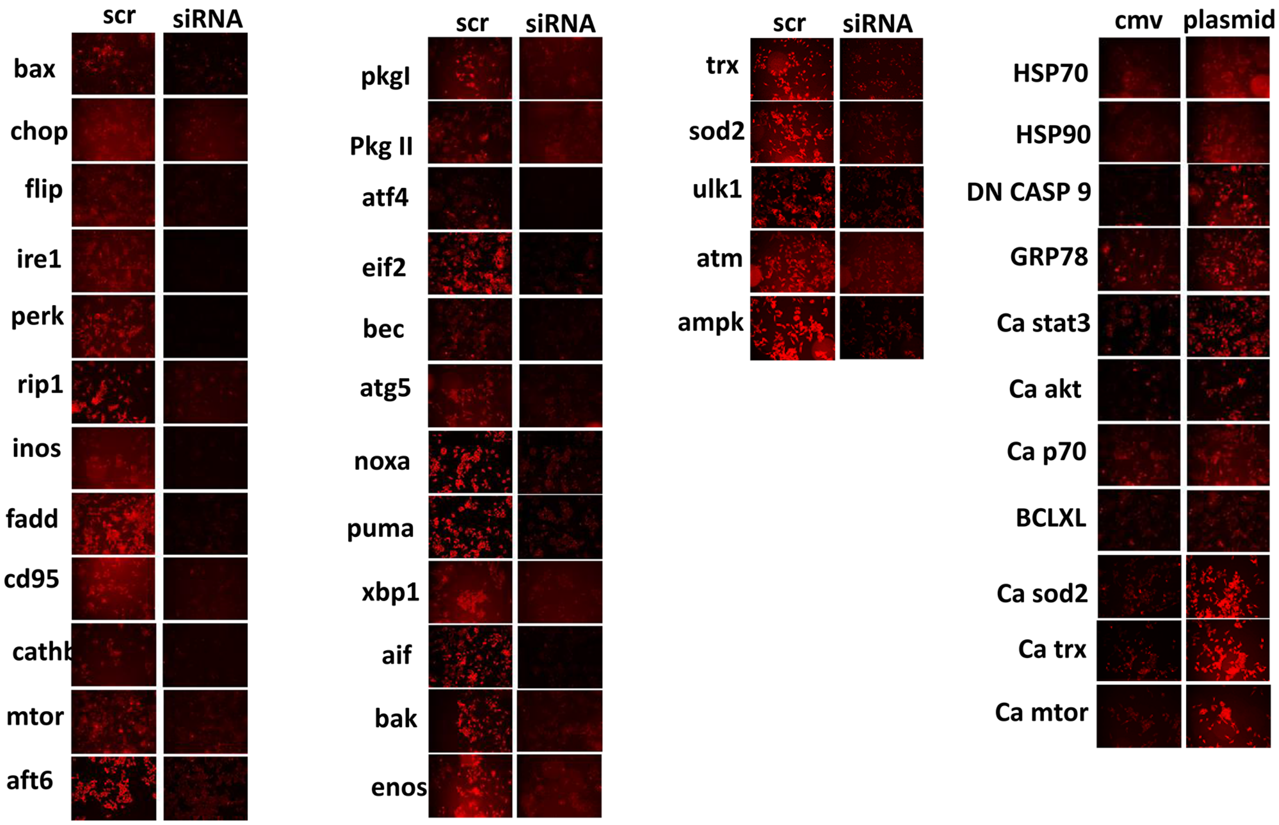

Supplementary Figure 13: Control siRNA knock down images and control over-expression images. HCT116 tumor cells were transfected with a scrambled siRNA (scr) or with an siRNA molecule, or with CMV of a given plasmid to knock down or over express the specified protein. Twenty-four h after transfection/infection, cells were fixed in place and protein expression determined.
